# Novel Antimicrobial Peptide Dendrimers with Amphiphilic Surface and Their Interactions with Phospholipids — Insights from Mass Spectrometry

**DOI:** 10.3390/molecules18067120

**Published:** 2013-06-18

**Authors:** Piotr Polcyn, Paulina Zielinska, Magdalena Zimnicka, Anna Troć, Przemysław Kalicki, Jolanta Solecka, Anna Laskowska, Zofia Urbanczyk-Lipkowska

**Affiliations:** 1 Institute of Organic Chemistry PAS, Kasprzaka Str. 44/54, Warsaw 01-224, Poland; 2 National Institute of Public Health–National Institute of Hygiene, Chocimska Str. 24, Warsaw 00-791, Poland

**Keywords:** dendrimers, peptides, amphiphilic surface, antimicrobial, high salt conditions, circular dichroism, phospholipids, ESI-MS, collision-induced dissociation (CID)

## Abstract

A series of new peptide dendrimers with amphiphilic surface, designed around a dendronized ornithine (Orn) core were synthesized and characterized by ESI-MS, ^1^H-, ^13^C- NMR, and CD spectrometry. An improved antimicrobial potency against *S. aureus* and *E. coli* was detected as a result of an increased charge, higher branching and variable lipophilicity of the residues located at the C-terminus. Minimal inhibitory concentration (MIC) values indicated that the selected dendrimers were not sensitive to the physiological concentration of Na^+^ and K^+^ ions (100 mM), but expressed reduced potency at 10 mM concentration of Mg^2+^ and Ca^2+^ ions. Circular dichroism (CD) curves measured under various conditions revealed structure and solvent-dependent curve evolution. ESI-MS studies of gas-phase interactions between selected dendrimers and both anionic (DMPG) and neutral (DMPC) phospholipids revealed the presence of variously charged dendrimer/phospholipid aggregates with 1:1 to 1:5 stoichiometry. The collision-induced fragmentation (CID) of the most abundant [dendrimer/phospholipid]^2^^+^ ions of the 1:1 stoichiometry demonstrated that the studied dendrimers formed stronger complexes with anionic DMPG. Both phospholipids have higher affinity towards dendrimers with a more compact structure. Higher differences in CID energy necessary for dissociation of 50% of the complex formed by dendrimers with DMPG *vs.* DMPC (∆CID_50_) correlate with a lower hemotoxicity. Mass spectrometry results suggest that for a particular group of compounds the ∆CID_50_ might be one of the important factors explaining selectivity of antimicrobial peptides and their branched analogs targeting the bacterial membrane. Both circular dichroism and mass spectrometry studies demonstrated that dendrimers of *N*^α^- and *N*^ε^-series possess a different conformation in solution and different affinity to model phospholipids, what might influence their specific microbicidal mechanism.

## 1. Introduction

The increasing resistance of microbial pathogens to conventional antibiotics has instigated strong worldwide efforts to develop antimicrobial agents with new structures and mechanism of action. Recently, several types of bactericides with dendrimeric structure have attracted significant attention, providing nanomolecules developed to enable novel therapeutic strategies [1]. Dendrimers have emerged as a new class of polymers, characterized by branched structure, high monodispersity and a high density of the surface groups. These properties encode their most common application as potential drug carriers, gene transfer devices and tools for imaging of biological systems [2]. Dendrimers like PAMAMs [3,4] PPIs [5], poly(l-lysine) [6], carbosilane [7] and viologen-phosphorus [8] *etc.*, carrying numerous quaternary amonium or alkylamonium groups at the surface, have been proposed as polycationic microbicides or carriers of antibiotic moieties [9]. They express a broad activity against pathogenic Gram-positive and Gram-negative bacteria, targeting mostly bacterial membranes. Even though this view has been somewhat revised lately, polycationic macromolecules deliver a brute force approach and need to be extensively modified to decrease the host cell toxicity [10]. On the other hand, the chemical versatility of dendrimers provides an opportunity to lower their toxicity and retain bactericidal potential by partial PEG-ylation [11], acetylation [12] or glycosylation [13].

Regarding the complex structure of the microbial envelope, *i.e.*, the Gram-specific combination of cell wall and cell membrane composition, we hypothesized that the modification of amphiphilic dendrimeric peptides might have beneficial effects on their biological activity. This concept led to the design of a novel class of amphiphilic, low molecular weight peptide dendrimers synthesized from orthogonally substituted amino acids containing basic (Lys, Arg) and lipophilic (Phe, Ala, Trp, *etc.*) side chains [14,15,16,17]. These novel amphiphiles of the 1st and 2nd generation, typically based on (Lys)Lys(Lys) dendrons, clearly different from polycationic antimicrobial dendrimers, expressed a significant potency against bacteria, including methicillin-susceptible and methicillin-resistant *Staphylococcus aureus* (MRSA), susceptible and extended spectrum b-lactamase (ESBL) variants of *Escherichia coli,* as well as antifungal activity against the *Candida* genus. Due to their properties, such dendrimers can be regarded as mimetics of natural, amphiphilic antimicrobial peptides (AMPs), designed by Nature as non-specific endogenous biocides (*i.e.*, less likely to develop resistance) [18]. Significance of amphiphilicity in construction of antimicrobial dendrimers has also been exploited by Kallenbach *et al.* who applied the (Lys)Lys(Lys) dendron to synthesize compounds containing four copies of the WR sequence (tryptophane- arginine) that inhibited the formation of *E. coli* RP437 biofilms [19]. Grinstaf *et al.* designed anionic Janus-type dendrimers, relatively active against the Gram-positive *B. subtilis*,but not harmful to human umbilical vein endothelial cells [20]. On the other side, recent studies on relatively small, di-, tri- and octacarboxyl dendritic amphiphiles revealed that even among less toxic, negatively charged branched amphiphiles, antimicrobial potency and level of cytotoxicity strongly depend on their 3D structure [21,22].

**Figure 1:**
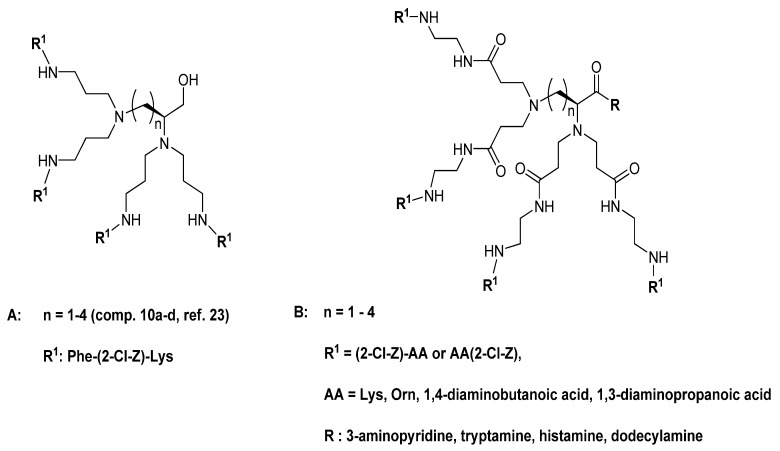
General structures of previously studied compounds **A** [23] and the present compounds **B**.

Recently, we have reported synthesis of group of dendrimers, originated from the new type of core molecules. We detected a higher antimicrobial potency than found previously in dendrimers based on the Lys-dendron [23]. Synthesis of the central fragment of dendrimers by applying the procedure developed by Voegtle *et al.* for obtaining the so-called “cascade molecules” [24] resulted in a partially resolved mixture of tris- and tetrakis-*N,N*′- cyanoethylated basic amino acids (Lys, Orn, *etc.*). Moreover, the attempted efficient hydrogenolysis of the tetrabranched core compounds required harsh conditions that simultaneously converted the C-terminal carboxyl to a hydroxymethyl group (Figure 1, structure **A**). It was evident from all previous studies on antimicrobial dendrimers that charge distribution and molecular topology contribute significantly to their biological profile. Therefore, synthetic efforts leading to the novel molecules and their biological evaluation seemed to be well justified. 

Herein, we present an efficient route leading to new tetra(octa)-branched dendrimers, by coupling the core molecules with orthogonally substituted basic amino acids, which offers an easy access to amphiphilic compounds with variable structure and lipophilicity (Figure 1, structure **B**). In contrast to di-block amphiphiles, constructed from hydrophilic/positively charged and lipophilic domains [20,21,22], the title dendrimers have an amphiphilic surface with a high degree of control over distances and relative orientations between cationic and lipophilic groups, as well as the arm lengths and charge distribution.

This group of dendrimers was tested *in vitro* using the microdilution broth technique against standard Gram-positive, methicillin susceptible and resistant strains of *Staphylococcus aureus*, and Gram-negative *Escherichia coli* and *Pseudomonas aeruginosa*. In addition their toxicity against red blood cells was examined. It is known that biologically relevant cations like Ca^2+^ and Mg^2+^ residing in cell envelope of Gram-negative genera often influence the microbicidal potency of peptides (e.g., AMPs). Thus, for the selected compounds, the minimal inhibitory concentration (MIC) against *E. coli* was assayed in the presence of increasing concentrations of divalent (Mg^2+^ and Ca^2+^) and monovalent (Na^+^ and K^+^) ions. A study of the conformational characteristics of the two isomeric groups of dendrimers in various media was performed using circular dichroism spectroscopy. 

While supramolecular factors affecting membranolytic properties of AMPs have been widely reviewed, the study of interaction of antimicrobial dendrimers with model phospholipids was mostly limited to the investigation of some commercial dendrimers [25,26,27,28]. In this work, mass spectrometry (ESI-MS) is proposed as a tool for studying, for the first time, the range of energetic factors of the dendrimer/phospholipid interactions in relation to the structure of dendrimers and character of phospholipids. Collision-induced fragmentation (CID) of the [dendrimer/phospholipid]^n^^+^ aggregates was performed for five dendrimers of different structure, lipophilicity and biological properties. As model phospholipids, the zwitterionic 1,2-dimyristoyl-glycero-3-phosphocholine (DMPC) and anionic 1,2-dimyristoyl-sn-glycero-3-phospho-*rac*-(1-glycerol) sodium salt (DMPG) were selected. 

This manuscript presents a facile synthetic route to the novel type of peptide dendrimers, folded into an amphiphilic surface. They expressed a high potency against reference a *E. coli* strain under normal as well as elevated ionic strength conditions. ESI-MS studies on gas phase interactions between five selected dendrimers and anionic (DMPG) and neutral (DMPC) phospholipids revealed that collision-induced fragmentation (CID) of the [dendrimer/phospholipid]^n^^+^ ions may provide important information concerning the influence of molecular structure of dendrimers on electrostatic interactions with model phospholipids. Further development of these compounds could potentially lead to an interesting alternative to the presently used antimicrobials. 

## 2. Results and Discussion

### 2.1. Synthesis

**Scheme 1:**
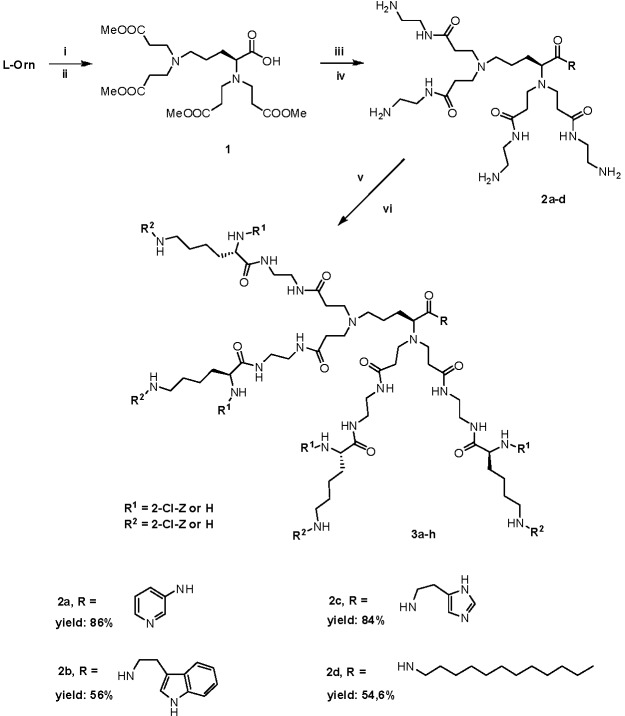
Synthesis of core molecules **2a**–**h** and final dendrimers **3a**–**h**.

Herein, we present the divergent method of synthesis of a group of dendrimers **3a**–**h**, built around the core molecules that originate from ornithine (Orn) and contain different residues located at the C-terminus (Scheme 1). Briefly, the basic amino acid ornithine (Orn) was subjected to the reaction sequence known from Tomalia’s work, *i.e.*, Michael addition of methyl acrylate to primary amines, followed by aminolysis with ethylenediamine [29]. Coupling of the C-terminus with 3-aminopyridine (3-AP), histamine (HisN), tryptamine (TrpN) and dodecylamine (ddN), yielded tetrabranched core molecules **2a**–**d**. Subsequent coupling of the core molecules with 10% excess of (2-Cl-Z)-Lys(Boc) or Boc-Lys(2-Cl-Z), afforded two isomeric series of dendrimers **3a**–**d**, and **3e**–**h**, respectively (Scheme 1). Dendrimers were prepared as water soluble hexahydrochlorides that melted within a relatively sharp temperature range when freshly lyophilized. Salts of compounds derived from *N*^ε^-substituted lysines also exhibited a higher melting points than salts derived from *N*^α^-lysines. The Supplementary Material associated with this article contains relevant MS, ^1^H- and ^13^C-NMR data, and ESI-MS spectra of dendrimer/phospholipid mixtures.

*Reagents and Conditions:* (i) CH_2_=CH-COOMe, 6 equiv. NaOH, 1eq, MeOH, 24 h, reflux; (ii) 1 equiv. HCl/MeOH, yield 67.7% (iii) **2a**, **2e**-3-aminopyridine, **2b**, **2f**-tryptamine, **2c**, **2g**-histamine, **2d**, **2h**-dodecylamine, 1.2 equiv, DCC, HOBt, DMF, 24 h; (iv) ethylenediamine, MeOH, 5 days, *n*-BuOH, and final dendrimers **3**: (v) **3a**, **3b**, **3c** and **3d**-4.4 eq (2-Cl-Z)-Lys(Boc), **3e**, **3f**, **3g** and **3h**-4.4 eq Boc-Lys(2-Cl-Z), DCC, HOBt, THF, 48 h (vi) TFA, HCl/EtOAc.

### 2.2. Microbiological and Hemolysis Studies of Dendrimers

**Figure 2:**
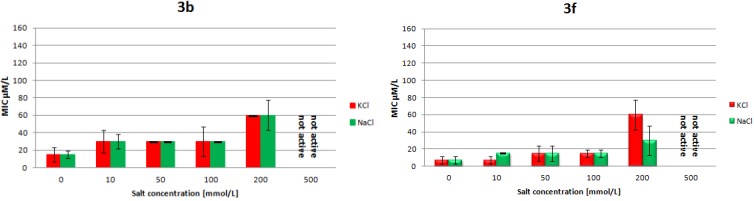
The effect of monovalent cations, Na^+^ or K^+^ on the MICs of **3b** and **3f** against *E. coli* ATCC 25922 (black bars represent mean error).

The results of the *in vitro* antimicrobial activity assay of amphiphilic dendrimers **3a**–**h** tested against two strains of Gram-positive *S. aureus*, Gram-negative *E. coli*, and *P. aeruginosa* by the conventional microdilution technique are shown in Table 1. All dendrimers have (+)6 charge spread along the scaffold with higher charge accumulation around the center of the compounds **3a**–**d**. The potency of analogs with tryptamine (compounds **3b**, **3f**) and dodecylamine (compounds **3d**, **3h**) residues located at the C-terminus is in the range characteristic for many natural antimicrobial peptides (0.5–20 µM). Of particular interest is compound **3d**, which is broadly active, including against methicillin-resistant strain of *S. aureus* ATCC 43300 (0.5 µM) and pathogenic *P. aeruginosa* (8 µM). The antimicrobial activity of many cationic AMPs is greatly affected by mono- and divalent cation concentrations, particularly in tests against Gram-negative bacteria. To assess the influence of salinity and chemical structure on efficacy, the diastereoisomeric pair of dendrimers **3b** and **3f** was tested in a MIC assay in the presence of physiological concentrations of the monovalent (Na^+^ and K^+^) and divalent (Mg^2+^ and Ca^2+^) cations. Both dendrimers were not sensitive to an increase of Na^+^ or K^+^ concentration up to 100 µM when tested against *E. coli* ATCC 25922. Moreover, they retained much activity at 200 µM concentration (Figure 2).

**Table 1:** Minimal inhibitory concentrations (MIC, µM) ^a^ exhibited by compounds **3a**–**h**^b^.

Strain	3a (3-AP)	3b (TrpN)	3c (HisN)	3d (ddN)	3e (3-AP)	3f (TrpN)	3g (HisN)	3h (ddN)
*S. aureus* ATCC 25923	2.85	1.87	24.7	1.85	16.6	0.93	51	3.7
*S. aureus* ATCC 43300	>252	12.1	141	0.46	142	20.4	16.4	1.85
*E. coli* ATCC 25922	>252	12.1	141	1.85	142	7.9	211	7.9
*P. aeruginosa* ATCC 27853	>252	51	141	7.8	142	60	211	32

^a^ MIC values of the reference antibiotic compounds: Penicillin G against *S. aureus* ATCC 25923-6.6 (µM); polymyxin B against *E. coli* ATCC 25922 and *P. aeruginosa* ATCC 27853-0.55 (µM); ^b^ CAMBH medium contains 0.5 mM of Ca^2+^ and Mg^2+^ and no K^+^ and Na^+^ ions.

**Figure 3:**
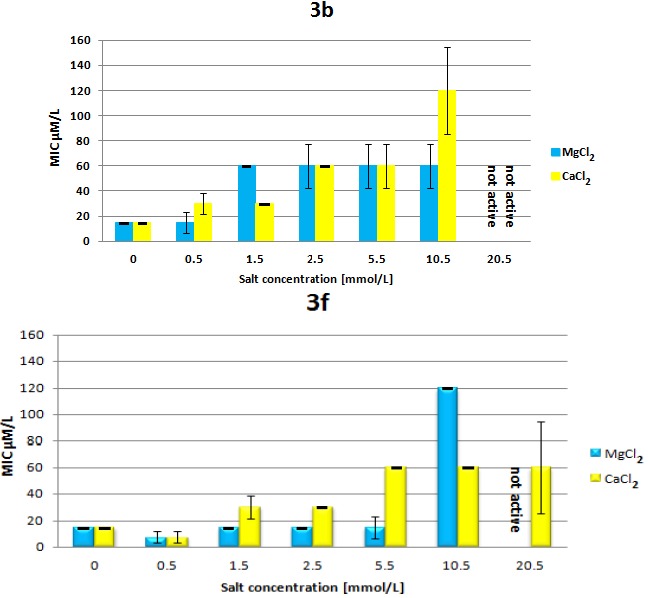
The effect of Ca^2+^ or Mg^2+^ cations on the minimum inhibitory concentration (MIC) of **3b** and **3f** against *E. coli* ATCC 25922 (black bars represent mean error).

The biologically relevant, divalent cations Ca^2+^ and Mg^2+^ known to maintain integrity of bacterial membranes, at higher concentrations lowered the antimicrobial potency of **3b** and **3f** in a structure-dependent manner (Figure 3). While the increase of Ca^2+^ and Mg^2+^ concentration up to a 5 mM induced a slow decrease of the bioactivity of both dendrimers, the sudden decrease is observed (MIC = 119.8 µM) for **3b** and **3f** when the respective Ca^2+^ and Mg^2+^ ions concentration reached 10.5 mM. 

**Figure 4:**
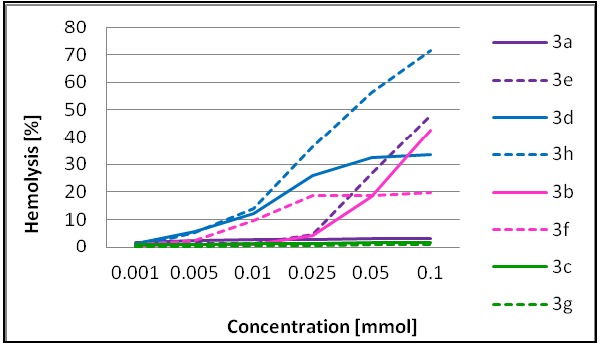
Hemolysis [%] induced by dendrimers on red blood cells suspended in PBS buffer (*N*^α^-series shown as continuous line, *N*^ε^-series shown as dotted line).

The hemolytic activity of cationic antimicrobial peptides against human erythrocytes is often used as a measure of their therapeutic potential. In the present study we demonstrate that the hemolytic properties are strongly dependent on the structure and potency of dendrimers (Figure 4). The highest hemotoxicity was detected for compounds of the *N*^ε^-series **3h** and **3e**, with dodecylamine and tryptamine residues located at the C-terminus, respectively, whereas a much lower toxicity was detected for the not particularly active isomeric pair **3c** and **3g** with histamine and for **3a** with a 3-aminopyridine residue. On the other hand, dendrimer **3d** of the *N*^α^-series (isomer of **3h**), with a broad activity against Gram-positive and Gram-negative bacteria, showed only *ca.* 35% hemolysis at 100 μM.

The above data indicate that bioactivity and hemolytic properties of the studied dendrimers are structure dependent. Moreover, although the clinical application of many analogs of AMPs is challenged by their inability to maintain their activity under the physiological salt concentrations [30,31], the activities of the studied simple dendrimers seem to be less cation-dependent. 

### 2.3. Circular Dichroism Spectroscopy Reveals Structure-Dependent Curve Evolution

Circular dichroism (CD) spectroscopy was used recently to study the conformation of large dendrimers containing fragments with well-preorganized secondary structures, e.g., dendrimers extended with polyproline chains [32] or possessing helical sequences [33]. It remains an open question whether small dendrimeric peptides are able to adopt any kind of defined structural pattern that is expected considering the chemical structure of analogous open chain peptides. 

**Figure 5:**
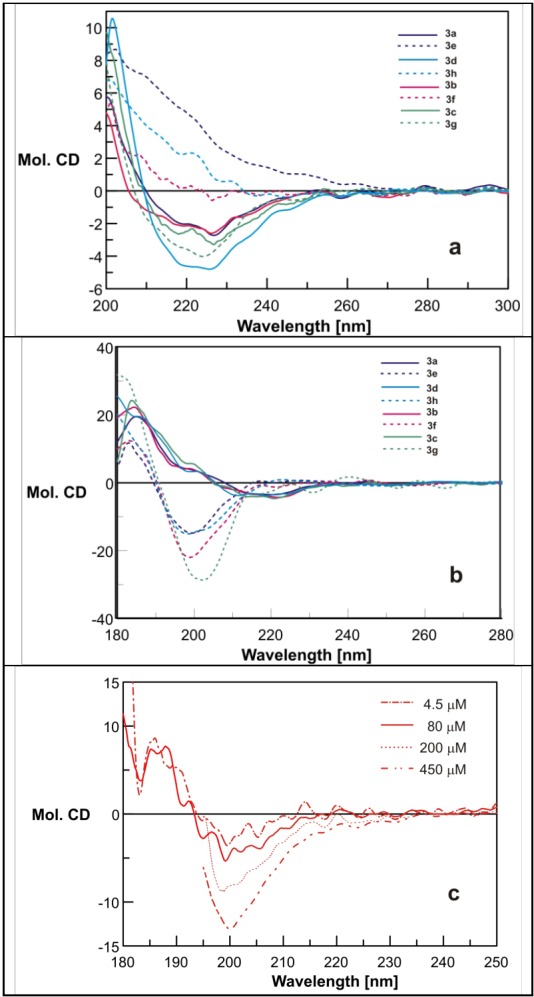
Molar ellipticity of dendrimers **3a**–**h**: (**a**) in MeOH, and (**b**) in water at peptide concentration ca 80 µM; (**c**) molar ellipticity of **3f** measured in 10 mmol PBS at various dendrimer concentration.

The results of our CD spectroscopy study of dendrimers **3a**–**h** in MeOH and in water solution (~80–90 µM) at room temperature are shown in Figures 5a,b. Two distinct types of spectra are detected for *N*^α^- (compounds **3a**–**d**) and *N^ε^-*substituted (compounds **3e**–**h**) derivatives. The CD spectra of the *N*^α^-series in methanol are characterized by presence of two main negative bands around 215–218 and 225–228 nm, while CD curves for those with four long arms are mostly located in the positive region. Interestingly, compound **3g**, with histamine residue at the C-terminus, that structurally belongs to the *N*^ε^-series, shows only in methanol a CD pattern closely matching that of the *N*^α^-series. In contrast, in water solution the CD spectra of the *N*^α^-series (Figure 5b) are located around the basal line, whereas the CD curves of the *N^ε^-*substituted derivatives show an intense deep minimum around 187–200 nm. Influence of both the PBS buffer and dendrimer concentration on the shape of the CD curves is significant. The CD spectrum of compound **3f** (Figure 5c, *N*^ε^-series), measured in 10 mmol PBS at pH 7.4, for 4.5 and 80 μM concentration shows the disappearance of shape and intensity compared to that measured in pure water (Figure 5b, red dotted line). The molecular ellipticity measured at 200 and 450 μM dendrimer concentration gradually acquired the shape and magnitude of that measured in water. On the other hand, the CD spectra for dendrimer **3c** (*N*^α^-series) measured in MeOH and in water also show concentration-dependency (see Figure S1). 

This is indicative of the presence of intermolecular interactions between the dendrimer molecules that at a higher dendrimer concentration lead to the formation of aggregates.

The gradual decrease of antimicrobial potency against *E. coli* observed at higher Ca^2+^ and Mg^2+^ ion concentration, observed for the two representative isomeric dendrimers **3b** and **3f** (Figure 3) might suggest that although already positively charged, these branched molecules may potentially interact with divalent cations. This hypothesis was further tested by following the CD spectra of **3b** and **3f** upon addition of 5, 10 and 20-fold molar excess of the respective Ca^2+^ and Mg^2+^ chlorides (see Figure S2). For a more branched dendrimer like **3b** a noticeable impact upon addition of Ca^2+^ and a much lower one on addition of Mg^2+^ ions is detected, whereas, for a more flexible tetrabranched dendrimer **3f**, the addition of Mg^2+^ ions is more significant. In all cases, a large shift in intensity upon addition of 5 molar equivalents of the respective salts suggests that the initial interactions might have an intramolecular character. The observed trends are in agreement with variations of the MIC’s measured at 10.5 mM concentration of Ca^2+^ and Mg^2+^chlorides (Figure 3).

In summary, circular dichroism methods were used to study conformations of dendrimers lacking a long sequence of amino acids, in different solvents and at different dendrimer concentrations. These studies show that, irrespective of the type of solvent, both groups of isomers adopt a different range of conformations. Molecular conformations of the selected amphiphilic dendrimers depend on several environmental factors, such as type of solvent, ionic strength and concentration, which strongly suggests their ability to aggregate, which is important for initial interactions between dendrimer and microbial membranes.

Since the available data supports the concept that the presence of the defined secondary structure of AMPs in solution is not determinative in the context of its activity, it is assumed that their biological activity depends rather on a synergistic interplay between positive charge and molecular structure and interactions with their biological targets, *i.e.*, biomembranes. The observation that the title dendrimers are biologically active irrespective of to which structural group they belong, shows similarity to the behavior of AMPs and suggests that various aspects of their interactions with biological membranes deserve to be studied in more detail.

### 2.4. Mass Spectrometry Studies of Gas Phase Complexation of Dendrimers by Phospholipids

Mass spectrometry (MS) is a recognized method for characterizing non-covalent interactions. Modern techniques, based on advanced mass-spectrometry, are regarded as a highly relevant physico-chemical tool to study various features of non-covalent complexes such as stoichiometry, dissociation constants, conformations and binding sites [34]. A number of published reviews and papers have defined the scope and limitations of mass spectrometry in the exploration of the most important issues of supramolecular chemistry [35,36,37]. The critical step in interpreting the results of MS experiments is to scrutinize the difference in the nature of non-covalent interactions in solution and in a gas phase. Going from solution to a solvent-free gas phase environment has a direct influence on the strength of interactions of molecules within non-covalent complex, e.g., the electrostatic forces are strengthened in vacuum, whereas the hydrophobic interactions become weaker. Taking into account the specificity of interactions between molecules in the gas phase, one can conclude that the most stable complexes in MS experiments are those in which the hydrogen bonding and electrostatic interactions play a dominant role in assembling the molecules. Other issues such as an entropic factor as well as the kinetic *vs.* thermodynamic stability should be kept in mind when any comparison is made between gas-phase and condensed-phase stability measurements of non-covalent complexes. The comparison of the results derived from measurements in the gas phase with those obtained in the solution may help assessing the type of bonding interactions that keep complexes together.

The unique composition of microbial and mammalian membranes is considered a source of selectivity of natural antimicrobial peptides and their mimics towards pathogenic species. The fact that the inner membrane of *E. coli* contains up to 27% of anionic phospholipids like DPPG and cardiolipins provides for the preferential electrostatic interactions with cationic amphiphilic peptides. While the chemical and structural factors that are responsible for the selectivity of natural antimicrobial peptides have been studied widely [38], the interactions of dendrimeric peptides with membrane phospholipids have seldom been discussed [39]. Extremely important for the life cycle, complex molecular processes, involving cell membranes are dynamic and very hard to follow, even utilizing simple models. For this reason, the application of mass spectrometry that allows to separate and study in detail certain molecular adducts seems to be an interesting alternative.

**Figure 6:**
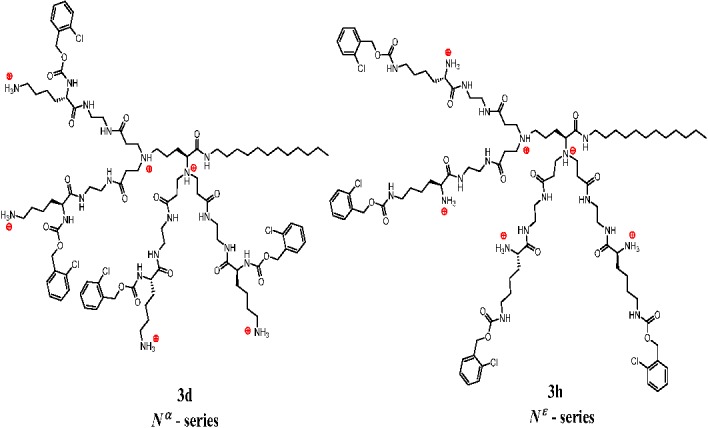
Molecular structure of isomeric **3d** and **3h** hexachlorides

In the present work we have studied the relative gas-phase stability of charged dendrimer/phospholipid complexes by applying collision-induced dissociation (CID). The following dendrimers were selected for complexation studies: three dendrimers of the *N*^α^-series—**3a**, **3c** and **3d**, and two dendrimers of the *N*^ε^-series—**3f** and **3h** (for stereochemistry see Figure 6). Both series have different structures in regard to the relative distances between cationic and lipophilic groups resulting in different access to the positively charged amino groups. As their model phospholipid partners, anionic 1,2-dimyristoyl-sn-glycero-3-phospho-*rac*-(1-glycerol) sodium salt (DMPG) and zwitterionic/neutral 1,2-dimyristoyl-glycero-3-phosphocholine (DMPC) were chosen.

All dendrimers have six cationic centers that might potentially bind phospholipids by electrostatic forces. In fact, in the gas phase DMPC and DMPG form charged aggregates with dendrimer/phospholipid ratios ranging from 1:1 to 1:5 (for DMPG), suggesting that the most crowded *N^α^* amino group is probably far less accessible for complexation (Figures S3–S6 show representative ESI-MS spectra of the dendrimer/phospholipid mixtures). 

The analysis of the fragmentation pathways of the non-covalent complexes formed between dendrimers and DMPC revealed that these complexes follow the same fragmentation pathway and dissociate into singly charged dendrimers and phospholipids (Supporting Information, Figure S7b). In contrast, the non-covalent complexes of DMPG undergo fragmentation into doubly charged dendrimers and neutral phospholipids (Supporting Information, Figure S7a). In both cases dendrimer-phospholipid complexes follow the same mechanism upon dissociation in which the charge separation occurs. 

**Figure 7:**
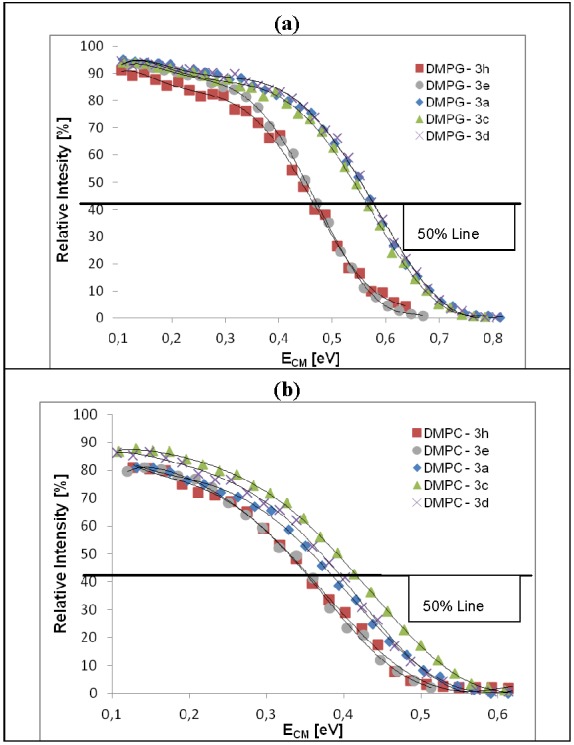
Dissociation efficiency curves of dendrimer/phospholipid [1:1]^2+^ complexes: (**a**) DMPG/**3h** (red square), DMPG/**3e** (grey circle), DMPG/**3c** (green triangle), DMPG/**3a** (blue square), DMPG/**3d** (cross) and (**b**) DMPC/**3h** (red square), DMPC/**3e** (grey circle), DMPC/**3c** (green triangle), DMPC/**3a** (blue square), DMPC/**3d** (cross). The *y* and *x* axis represents the relative intensity of a complex in proportion to the sum of the intensities of the fragment ions and center-of mass collision energy under single collision conditions, respectively. Collision energies for 50% dissociation of the complexes (50% Line) are as follows: 0.44 (DMPG/**3h**), 0.45 (DMPG/**3e**), 0.54 (DMPG/**3c**), 0.55 (DMPG/**3a**), 0.55 (DMPG/**3d**), 0.33 (DMPC/**3h** and DMPC/**3e**), 0.39 (DMPC/**3c**), 0.36 (DMPC/**3a**) and 0.37 (DMPC/**3d**).

The relative stability of dendrimer/phospholipid complexes was established based on the dissociation efficiency curves (plots of normalized intensity of complexes *versus* center-of-mass collision energy under single collision conditions). The collision energy, required for the half-dissociation of complex (CID_50_) was used as a measure of complex stability (Figure 7).

The following observations are apparent from the plots:

- Dendrimers **3e** and **3h** of the *N*^ε^-series form less stable complexes with both phospholipids; however complexes with DMPG molecules, bearing formal negative charge are more stable than that with zwitterionic DMPC by 0.11 eV.
- DMPG molecules form complexes with similar gas phase stability with dendrimers of the *N*^α^-series: **3a**, **3c** and **3d**; however these complexes are more stable than DMPG complexes with **3e** and **3h** by ca. 0.1 eV. Similar differences in binding strength between DMPG and DMPC have been reported for peptide-phospholipid non-covalent complexes [40].
- Compounds of the *N*^α^- and *N*^ε^-series show different recognition patterns with DMPG *vs.* DMPC in the gas phase. This accounts for the significant difference in stability of complexes for a particular dendrimer, as evidenced by ∆CID_50_ values reported in Table 2. Moreover, a high ∆CID_50_ values correlate with a lower level of hemotoxicity (shown in Figure 4).

**Table 2:** ΔCID_50_ values (eV) and level of hemotoxicity exhibited by selected dendrimers.

Dendrimer	ΔCID_50_ (eV)	Hemotoxicity
**3h**	0.11	high
**3e**	0.12	high
**3c**	0.15	low
**3a**	0.19	low
**3d**	0.18	low

The results obtained from the MS experiments clearly show that the gas phase stability of the complexes is directly related to their molecular structure, *i.e.*, charge distribution, arm length and hydrophobicity of the dendrimer, as well as the character of phospholipids. Both phospholipids form weaker complexes with dendrimers of high molecular flexibility (long flexible arms of the *N*^ε^-series) and stronger complexes with molecules characterized by a more compact structure and charges located on the surface, such as the *N*^α^-series dendrimers. The effect of chemical structure of a phospholipid on its biophysical interactions with dendrimers is reflected by different separation between half-dissociation points of dendrimers **3e** and **3h** and family **3a**, **3c** and **3d**: ca 0.11 and 0.02 eV, respectively for DMPG and DMPC.

In summary, it appears that the difference between relative stability of complex formed by a particular dendrimer with DMPG *vs.* DMPC, as quantified by large ∆CID_50_ value, reflects not only aspects of gas-phase electrostatic attraction between phospholipids and dendrimer, but also involves more subtle effects like dendrimer hydrophobicity, charge distribution and molecular flexibility. Therefore, these factors may account for a higher affinity to the negatively charged phospholipids, *i.e.*, it points out to higher selectivity towards prokaryotic cells. Although the above data are preliminary, the apparent correlation of a high ∆CID_50_ with a lower hemotoxicity suggests that such an approach might give valuable information during development of membrane-active low toxic compounds.

## 3. Experimental

### 3.1. General

All solvents and reagents were of analytical grade and were used without further purification. Mass spectra were recorded with a Mariner ESI time-of-flight mass spectrometer (PerSeptive Biosystems) for the samples prepared in MeOH. The ^1^H-NMR and ^13^C-NMR spectra were recorded using a Bruker Avance spectrometer at 500/125 or 400/100 MHz, respectively, using deuterated solvents and TMS as an internal standard. Chemical shifts are reported as δ values in parts per million, and coupling constants are given in hertz. The optical rotations were measured with JASCO J-1020 digital polarimeter. Melting points were recorded on a Köfler hot-stage apparatus and are uncorrected. Thin layer chromatography (TLC) was performed on aluminum sheets with silica gel 60 F_254_ from Merck. Column chromatography (CC) was carried out using silica gel (230–400 mesh) from Merck or Sephadex LH20. The TLC spots were visualized by treatment with 1% alcoholic solutions of ninhydrin and heating.

### 3.2. Synthesis and Characterization

#### 3.2.1. Preparation of *N,N*′-Tetrakis(methoxycarbonylethyl)-l-ornithine (1)

To a suspension of ornithine monohydrochloride (0.1 mol) in MeOH (150 mL), NaOH and (2 g, 0.2 mol) and methyl acrylate (51.65 g, 54 mL, 0.6 mol,) were added. The mixture was stirred for 48 h at reflux and then cooled and evaporated *in vacuo*. The oily product was shaken with acetone (300 mL) and then with 2M HCl in MeOH (100 mL). The solution was filtered and evaporated to dryness. The product was purified by flash chromatography (SiO_2_) eluting with an 8:2 mixture of EtOAc and hexane plus 5% methanol, yielding 28.3 g (66.7%) the *N,N′*-tetrakis(methoxycarbonylethyl)-L-ornithine (**1**), as a pale yellow oil. MSLR (ESI, MeOH**):** 477 (M+H^+^), 499 (M+Na^+^). ^1^H-NMR (400 MHz, CDCl_3_) δ 1.55, 1.77 (2 m, 4 H, β, γCH_2_), 2.51 (bm, 10 H, C***H***_2_COOMe, δCH_2_), 2.85, 2.95 (2 m, 8 H, α, δN-C***H***_2_-C), 3.27 (m, 1H, αCH), 3.67 (2 s, 12 H, -OCH_3_). ^13^C-NMR δ 23.9 (γC), 26.7 (βC), 30.9, 33.6 (δ, αC***H***_2_COOMe), 46.9, 48.2 [α, δN-(CH_2_)_2_], 51.6, 51.7 (-OCH_3_), 53.3 (δC), 63.9 (αC), 172.5, 172.8 (COOMe),175.5 (COOH). Anal. calcd**.** for C_21_H_36_O_10_N_2_: C, 52.93; H, 7.61; N, 5.87. Found: C, 52.83; H, 7.63; N, 5.77. [α]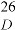: −32.6 ± 1° (*c* 2, acetone).

#### 3.2.2. Preparation of (3-Aminopyridyl)-amide (**1a**)

Compound **1** (4.5 g, 10 mmol) and 3-aminopyridine (1.4 g, 20 mmol) were dissolved in dry pyridine (30 mL) and cooled to −15 °C. Next, slowly and with a very intensive stirring POCl_3_ (1 mL, 11 mmol) was added to the solution. After 1 h the solvent was removed, EtOAc (100 mL) was added and the mixture was washed consecutively with 10% Na_2_CO_3_, H_2_O, 1% citric acid and brine (100 mL each). The organic layer was dried over anhydrous Na_2_SO_4_, filtered and then evaporated *in vacuo* to dryness. The residue was purified by flash chromatography (EtOAc-hexane 7:3 + 3% MeOH) to give 4.8 g (86.8%) of **1a** as an yellow oil. C_26_H_40_N_4_O_9_, MSLR (ESI, MeOH): 553 (M+H^+^), 675 (M+Na^+^). ^1^H-NMR (500 MHz, CDCl_3_) δ 1.5–1.9 (3 m, 4 H, γ, βCH_2_), 2.45 (bm, 10 H, C***H***_2_COOMe, δCH_2_), 2.87, 2.94 (2 m, 8H, α, δN-C***H***_2_-C), 3.35 (dd, *J* 2.2, 5.4 Hz, 1 H, αCH), 3.63, 3.66 (2 s, 12 H, -OCH_3_), 7.30 (m, 1 H, C_5_H *AP*), 8.31 (m, 2 H, C_4_H, C_6_H *AP*), 8.77 (s, 1 H, C_2_H *AP*). ^13^C-NMR δ 23.4 (γCH_2_), 25.8 (βCH_2_), 32.5, 33.1 (δ, α***C***H_2_COOMe), 46.7, 49.2 [α, δN-(CH_2_)_2_], 51.6, 51.8 (-OCH_3_), 53.7 (δC), 65.4 (αC), 123.4, 126.2, 135.5, 141.2, 144,6 (C_5_, C_4_, C_3_, C_2_, C_6_*AP*), 173.1 (CONH), 172.9, 173.0 (COOMe).

#### 3.2.3. General Procedure for Preparation of Tryptamide (**1b**), Histamide (**1c**) and Dodecylamide (**1d**)

To a stirred DMF solution (30 mL) containing **1** (4.76 g, 10 mmol), tryptamine (3.2 g, 20 mmol), HOBt monohydrate (1.54 g, 10 mmol) and DCC (2.1 g, 10.17 mmol) were added. The mixture was stirred at room temperature for 24 h, DCU was filtered off and solvent was evaporated *in vacuo.* The oily residue was dissolved in ethyl acetate (150 mL) and washed consecutively with 10% Na_2_CO_3_, H_2_O, 1% citric acid (3 times) and brine (150 mL each). The organic phase was dried over MgSO_4_ overnight, filtered and evaporated to dryness. The residue was purified by flash chromatography (EtOAc-hexane 7:3 + 3% MeOH) to give 5.3 g (85.8%) of **1b** as dark orange oil. C_31_H_46_N_4_O_9_, MSLR (ESI, MeOH**):** 619 (M+H^+^), 641 (M+Na^+^). ^1^H-NMR (500 MHz, CDCl_3_) δ 1.3–1.8 (4m, 4H, γ, βCH_2_), 2.31, 2.43 (2 bm, 10 H, C***H***_2_COOMe, δCH_2_), 2.76 (bm, 8 H, α, δN-C***H***_2_-C), 3.0 (t, *J* 6.9 Hz, 2 H, CH_2_-Ar *trNH*), 3.07 (dd, *J* 2.3, 5.4 Hz, 1 H, αCH), 3.55–3.7 (m, 14 H, -C***H***_2_-NH *trNH*, -OCH_3_), 7.04 (s, 1 H, C_1_*trNH*), 7.09, 7.16 (2 m, 2 H, C_5_, C_6_*trNH*), 7.34 (d, *J* 8.1 Hz, 1 H, C_7_*trNH*), 7.61 (d, *J* 7.9 Hz, 1 H, C_4_*trNH*). ^13^C-NMR δ 24.5 (γCH_2_), 25.1 (***C***H_2_-Ar *trNH*), 25.6 (βCH_2_), 32.4, 33.4 (δ, α***C***H_2_COOMe), 39.3 (-***C***H_2_-NH *trNH*), 46.4, 49.0 [α, δN-(CH_2_)_2_], 51.5 (-OCH_3_), 53.6 (δC), 64.7 (αC), 111.1, 113.0, 118.7, 119.1, 121.9, 122.0, 127.5, 136.4 (C_7_, C_2_, C_4_, C_5_, C_6_, C_1_, C_3_, C_8_*trNH*), 172.7, 172.9 (COOMe), 173.1 (CONH).

*Compound **1c***. Substrates: **1** (4.76 g, 10 mmol), histamine (C_5_H_9_N_3_, 1.2 g, 10,8 mmol). Yield: 4.9 g (86%), pale yellow oil. C_26_H_43_N_5_O_9_, MSLR (ESI**,** MeOH**):** 258 (M+2H^+^)^2^^+^, 570 (M+H^+^), 592 (M+Na^+^). ^1^H-NMR (500 MHz, CDCl_3_) δ 1.4–1.9 (4 m, 4 H, γ, βCH_2_), 2.42 (m, 10 H, C***H***_2_COOMe, δCH_2_), 2.69–2.86 (bm, 10 H, α, δN-C***H***_2_-C, CH_2_-Ar *hiN*), 3.12 (dd, *J* 2.0, 5.6 Hz, 1H, αCH), 3.53 (m, 2H, CH_2_NH *hiN*), 3.66 (s, 12 H, -OCH_3_), 6.82 (s, H, C_4_H *hiN*), 7.56 (s, 1 H, C_2_H *hiN*). ^13^C-NMR δ 24.5 (γCH_2_), 25.3 (βCH_2_), 26.8 (***C***H_2_-Ar *hiN*) 32.4, 33.4 (δ, α***C***H_2_COOMe), 38.7 (CH_2_NH *hiN*), 46.3, 49.0 [α, δN-(CH_2_)_2_], 51.4, 51.5 (-OCH_3_), 53.5 (δC), 64.5 (αC), 117.7, 134.7, 137.2 (C_4_, C_2_, C_5_*hiN*), 172.2, 173.0 (COOMe), 173.2 (CONH).

*Compound **1d***. Substrates: **1** (4.76 g, 10 mmol), dodecyloamine (C_12_H_27_N, 3.7g, 20 mmol). Yield: 3.51 g (54.6%), pale yellow oil. C_33_H_61_N_3_O_9_, MSLR (ESI, MeOH): 644 (M+H^+^), 666 (M+Na^+^). ^1^H-NMR (500 MHz, C_6_D_6_) δ 0.88 (t, *J* 6.9 Hz, 3 H, -CH_3_*dda*), 1.2–1.9 (4 bm, 24 H, C_2_H-C_11_H *dda*, γ, βCH_2_), 2.43 (m, 10 H, C***H***_2_COOMe, δCH_2_), 2.75, 2.84 (2 m, 8 H, α, δN-C***H***_2_-C), 3.11 (dd, *J* 2.1, 5.5 Hz, 1 H, αCH), 3.21 (m, 2 H, CH_2_NH *dda*), 3.65, 3.67 (2 s, 12 H, -OCH_3_). ^13^C-NMR δ 14.0 (C_12_*dda*), 22.6 (C_11_*dda*), 24.2 (γCH_2_), 25.5 (βCH_2_), 26.9, 27.0 (C_10_, C_3_*dda*), 29.3–29.6 (C_4_-C_9_*dda*), 31.2 (C_2_*dda*), 32.4, 33.5 (δ, α***C***H_2_COOMe), 39.2 (CH_2_NH *dda*), 46.5, 49.1 [α, δN-(CH_2_)_2_], 51.4, 51.5 (-OCH_3_), 53.6 (δC), 64.5 (αC), 173.7, 172.8 (COOMe), 173.0 (CONH).

#### 3.2.4. General Procedure for Preparation of Core Molecules **2a–d**

The respective amide **1a**–**d** (5 mmol) was dissolved in MeOH (20 mL) and added slowly to a mixture of ethylenediamine (22.3 g, 25 mL) and MeOH (50 mL) cooled to 0 °C. The reaction mixture was stirred at room temperature for 5 days. The solvent was evaporated to dryness. The residue was mixed with *n*-BuOH (20 mL) and evaporated four times to remove excess of ethylenediamine. The residue was evaporated for 6 h yielding the respective compounds **2a**–**d** characterized by MS and used directly for dendrimer preparation.

*Compound **2a***. Yield: 99%, yellow oil. MSLR (ESI**,** MeOH**):** 665 (M+H^+^), 687 (M+Na^+^). ^1^H-NMR (400 MHz, DMSO) δ 1.2–1.8 (3 bm, 4 H, γ, βCH_2_), 2.0–2.4 (2 bm, 10 H, C***H***_2_CONH, δCH_2_), 2.5–3.1 (3 bm, 24 H, α, δN-C***H***_2_-C, HN-C***H***_2_-C***H***_2_-NH), 3.35 (m, 1 H, αCH), 7.34 (m, 1 H, C_5_H *AP*), 7.9–8.1 (2 m, 8 H, NH_2_). 8.13 (m, 1 H, C_4_H *AP*), 8.25 (dd, *J* 1.5, 4.6 Hz, C_6_H *AP*), 8.82 (d, *J* 2.4 Hz, 1H, C_2_H *AP*). ^13^C-NMR δ 24.3, 25.1 (γ, βC), 33.1, 34.8 (α, δ***C***H_2_CONH), 41.3, 42.3 (NH-***C***H_2_-***C***H_2_-NH), 46.9, 49.5 [α, δN-(CH_2_)_2_], 52.8 (δC), 64.0 (αC), 123.5, 126.1, 135.6, 141.0, 144.0 (C_5_, C_4_, C_3_, C_2_, C_6_*AP*), 171.4, 171.5 (***C***ONH-(CH_2_)_2_-NH_2_), 172.4 (*Orn* CONH).

*Compound **2b***. Yield: 99%, yellow oil. MSLR (ESI, MeOH): 731 (M+H^+^), 753 (M+Na^+^). ^1^H-NMR (400 MHz, DMSO) δ 1.2–1.8 (2 bm, 4 H, γ, βCH_2_), 2.2–2.4 (2 m, 10 H, C***H***_2_CONH, δCH_2_), 2.5–3.1 (3 bm, 26 H, α, δN-C***H***_2_-C, HN-C***H***_2_-C***H***_2_-NH, CH_2_-Ar *trNH*), 3.2 (m, 1 H, αCH), 3.36 (m, 2 H, -C***H***_2_-NH *trNH*), 6.95, 7.06, 7.15, 7.32, 7.54 (5 m, 5 H, C_1_, C_5_, C_6_, C_7_, C_4_*trNH*), 7.95 (m, 8H, NH_2_). ^13^C-NMR δ 23.9 (γC), 25.3 (***C***H_2_-Ar *trNH*), 26.0 (βC), 33.3, 34.8 (δ, α***C***H_2_CONH), 39.0 (-***C***H_2_-NH *trNH*), 41.3, 42.1 (NH-***C***H_2_-***C***H_2_-NH), 46.8, 49.6 [α, δN-(CH_2_)_2_], 52.8 (δC), 63.2 (αC), 111.3, 111.8, 118.1, 118.3, 120.8, 122.6, 127.2, 136.2 (C_7_, C_2_, C_4_, C_5_, C_6_, C_1_, C_3_, C_8_*trNH*), 171.4, 171.5 (***C***ONH-(CH_2_)_2_-NH_2_), 171.9 (*Orn* CONH).

*Compound **2c***. Yield: 99%, yellow oil. MSLR (ESI**,** MeOH**):** 704 (M+Na^+^). **^1^**H-NMR (400 MHz, DMSO) δ 1.2–1.8 (2 bm, 4 H, γ, βCH_2_), 2.0–2.4 (2 bm, 10 H, C***H***_2_CONH, δCH_2_), 2.5–3.1 (3 bm, 26 H, α, δN-C***H***_2_-C, HN-C***H***_2_-C***H***_2_-NH, CH_2_-Ar *hiN*), 3.2 (m, 1 H, αCH), 3.4 (m, 2 H, CH_2_NH *hiN*), 6.78 (s, H, C_4_H *hiN*), 7.51 (s, 1 H, C_2_H *hiN*). ^13^C-NMR δ 23.9 (γCH_2_), 25.9 (βCH_2_), 27.0 (***C***H_2_-Ar *hiN*), 33.2, 34.8 (α, δ***C***H_2_CONH), 38.5 (CH_2_NH *hiN*), 41.3, 42.0 (NH-***C***H_2_-***C***H_2_-NH), 46.8, 49.5 [α, δN-(CH_2_)_2_], 52.8 (δC), 63.2 (αC), 116.8, 134.7, 136.9 (C_4_, C_2_, C_5_*hiN*), 171.5 (***C***ONH-(CH_2_)_2_-NH_2_), 171.9 (*Orn* CONH).

*Compound **2d***. Yield: 99%, yellow oil. MSLR (ESI, MeOH): 796 (M+H_2_O+Na^+^), 818 (M-H^+^+2Na^+^+H_2_O). ^1^H-NMR (400 MHz, DMSO) δ 0.88 (t, *J* 6.9 Hz, 3 H, -CH_3_*dda*), 1.2–1.9 (4 bm, 24 H, C_2_H-C_11_H *dda*, γ, βCH_2_), 2.1–2.4 (2 m, 10 H, C***H***_2_CONH, δCH_2_), 2.5–3.0 (2 bm, 24 H, α, δN-C***H***_2_-C, HN-C***H***_2_-C***H***_2_-NH), 3.1–3.3 (bm, 3 H, αCH, CH_2_NH *dda*). **^1^**^3^C-NMR δ 14.0 (C_12_*dda*), 22.1 (C_11_*dda*), 24.5 (γCH_2_), 25.3 (βCH_2_), 26.4 (C_10_, C_3_*dda*), 28.7–29.1 (C_4_-C_9_*dda*), 31.3 (C_2_*dda*), 33.2, 34.9 (α, δ***C***H_2_CONH), 38.2 (CH_2_NH *dda*), 41.1, 41.8 (NH-***C***H_2_-***C***H_2_-NH), 46.8, 49.5 [α, δN-(CH_2_)_2_], 52.8 (δC), 63.0 (αC), 171.5 (***C***ONH-(CH_2_)_2_-NH_2_), 171.6 (*Orn* CONH).

#### 3.2.5. General Procedure for Preparation of Dendrimers **3a**–**3h**

The respective core compound (**2a**–**2d,** 1.86 g, *ca.* 2.5 mmol) was dissolved in DMF (20 mL). To the obtained mixture, a solution of (Boc)-l-Lys(2-Cl-Z) or (2-Cl-Z)-l-Lys(Boc), (4.56 g, 11mmol), HOSu (1.26 g, 11 mmol) and DCC (2.31 g, 11 mmol) in THF (20 mL)as then added and the mixture was stirred for 48 h at room temperature. The DCU was then filtered off and the solvent was evaporated *in vacuo*. The residue was dissolved in EtOAc (150 mL) and washed consecutively with 10% aqueous Na_2_CO_3_, water, 1% citric acid solution (150 mL of each), dried over MgSO_4_, filtered and evaporated. The raw dendrimeric compound was purified by column chromatography using Sephadex LH-20 packing and MeOH as eluent, to yield pure compound as an amorphous foam. Then the dendrimers were converted to their corresponding hexahydrochlorides by deprotection of Boc-groups with TFA and replacement of CF_3_COO^−^ with Cl^−^ using of HCl-saturated EtOAc and drying over P_2_O_5_, to yield the respective dendrimers **3a**–**3h** as slightly hygroscopic, amorphous powders.

*Dendrimer **3a***. C_86_H_124_O_17_N_20_Cl_4_·7HCl, M = 2,107.0 g/mol (monoisotopic mass of unprotonated compound–1,848), yellow-white hygroscopic foam. Yield 1.1 g (70%). MSLR (ESI, MeOH**)**: 617 (M+3H^+^*main signal*), 925 (M+2H^+^). ^1^H-NMR (400 MHz, DMSO, 298K) δ 1.34, 1.4, 1.7 (3 m, 24 H, γ, δ, βCH_2_*Lys-branches*), 1.92, 2.1 (3 m, 4 H, *core* γ, βCH_2_), 2.7, 2.85 (2 m, 8 H, *core* C***H***_2_CONH), 2.9-3.6 (4 m, 34 H, εCH_2_*Lys-branches*, α, δN-C***H***_2_-C, HN-C***H***_2_-C***H***_2_-NH *and core* δCH_2_), 3.7 (m, 4 H, αCH *Lys-branches*), 4.5 (m, 1 H, *core* αCH), 5.1 (4 s, 8 H, Ar-C***H***_2_O), 7.28–7.35 (m, 8 H, C_4_H, C_5_H *2-Cl-Z*), 7.39-7.46 (m, 8 H, C_3_H, C_6_H *2-Cl-Z*), 7.90 (dd, *J* 5.7, 2.4 Hz, 1 H C_5_H *AP*), 8.60 (d, *J* 5.16 Hz, 1 H, C_4_H *AP*), 8.64 (m, C_6_H *AP*), 9.23 (s, 1 H, C_2_H *AP*). ^13^C-NMR δ 19.2 (*core* γC), 21.1 (γC *Lys-branches*), 24.6 (*core* βC), 28.5 (δC *Lys-branches*), 30.1 (βC *Lys-branches*), 29.4, 30.3 (*core* δ, α***C***H_2_CONH), 38.1 (εC *Lys-branches*), 40.0 (*core* NH***C***H_2_***C***H_2_NH), 48.2, 49.2 [*core* α, δN-(CH_2_)_2_], 51.3 (*core* δC), 52.2 (αC *Lys-branches*), 62.1 (Ar-***C***H_2_O), 63.6 (*core* αC), 126.2 (C_5_*AP*), 126.9, 128.9, 129.2, 129.3, 131.9 (C_5_, C_6_, C_4_, C_3_, C_2_*2-Cl-Z*), 134.0, 134.3 (C_6_, C_2_, *AP*), 134.4 (C_1_*2-Cl-Z*), 136.6, 138.1 (C_3_, C_4_*AP*), 155.5 (O-CO-NH), 168.1 (CONH *Lys-branches*), 168.5 (*core* CH_2_***C***ONH), 169.0 (*core* CONH).

*Dendrimer **3b***. C_91_H_130_O_17_N_20_Cl_4_·6HCl, M = 2,136.7 g/mol (monoisotopic mass of unprotonated compound–1,914), brown hygroscopic foam. Yield 1.1g (73%). MSLR (ESI, MeOH**)**: 639 (M+3H^+^), 653.667 (M+H^+^+2Na^+^*main signal*), 958 (M+2H^+^), 980 (M+2Na^+^). ^1^H-NMR (400 MHz, DMSO, 298K) δ 1.3-1.75 (bm, 28 H, γ, δ, βCH_2_*Lys-branches*; *core* β, γ, δCH_2_), 2.1–2.5 (2 m, 10 H, C***H***_2_CONH, *core* εCH_2_), 2.6–3.0 (2 m, 8 H, α, εN-C***H***_2_-C), 3.05–3.3 (bm, 2 H, εCH_2_*Lys-branches*; *core* HN-C***H***_2_-C***H***_2_-NH, CH_2_-Ar *trNH*), 3.4 (m, 3 H, -C***H***_2_-NH *trNH*, αCH), 3.93 (m, 4 H, αCH *Lys-branches*), 5.1-5.17 (4 s, 8 H, Ar-C***H***_2_O), 6.96, 7.04, 7.12 (3 m, 3 H, C_1_, C_5_, C_6_*trNH*), 7.30–7.37 (bm, 9 H, C_4_H, C_5_H *2-Cl-Z*, C_7_*trNH*), 7.40–7.51 (2 bm, 8 H, C_3_H, C_6_H *2-Cl-Z*), 7.56 (1 m, 1 H, C_4_*trNH*). ^13^C-NMR δ 22.3 (γC *Lys-branches*), 24.0 (*core* γC), 25.1 (***C***H_2_-Ar *trNH*), 25.7 (*core* βC), 26.7 (*core* δC), 28.9 (δC *Lys-branches*), 31.3 (βC *Lys-branches*), 33.1, 34.3 (*core* ε, α***C***H_2_CONH), 38.0, 38.1 (εC *Lys-branches*), 39.1 (-***C***H_2_-NH *trNH*), 39.3 (*core* NH***C***H_2_***C***H_2_NH), 46.7, 49.1 [*core* α, εN-(CH_2_)_2_], 51.1 (*core* εC), 54.5 (αC *Lys-branches*), 62.2 (Ar-***C***H_2_O), 63.6 (*core* αC), 111.0, 111.7, 117.8, 117.9, 120.6, 122.1 (C_7_, C_2_, C_4_, C_5_, C_6_, C_1_*trNH*), 126.7 (C_5_*2-Cl-Z*), 127.0 (C_3_*trNH*), 128.8, 129.0, 129.1, 131.8, 134.1 (C_6_, C_4_, C_3_, C_2_, C_1_*2-Cl-Z*), 136.1 (C_8_*trNH*), 155.2 (O-CO-NH), 170.8, 171.0 (*core* CH_2_***C***ONH), 171.4 (CONH *Lys-branches*), 171.8 (*core* CONH).

*Dendrimer **3c***. C_86_H_127_O_17_N_21_Cl_4_·7HCl, M = 2,124.1 g/mol (monoisotopic mass of unprotonated compound – 1,865), yellow-white hygroscopic foam. Yield 1.1g (64%). MSLR (ESI, MeOH): 622.667 (M+3H^+^, *single signal*). ^1^H-NMR (400 MHz, DMSO, 298K) δ 1.3–1.7 (bm, 28 H, γ, δ, βCH_2_*Lys-branches*; *core* β, γ, δCH_2_), 2.1–2.4 (2 m, 10 H, C***H***_2_CONH, *core* εCH_2_), 2.5–2.9 (3 m, 10 H, α, εN-C***H***_2_-C, CH_2_-Ar *hiN*), 3.0–3.4 (2 bm, 27 H, εCH_2_*Lys-branches*; *core* HN-C***H***_2_-C***H***_2_-NH, *core* αCH, CH_2_NH *hiN*), 3.93 (m, 4 H, αCH *Lys-branches*), 5.08–5.18 (4 s, 8 H, Ar-C***H***_2_O), 6.79 (s, 1 H, C_4_H *hiN*), 7.30–7.37 (m, 8 H, C_4_H, C_5_H *2-Cl-Z*), 7.4–7.5 (m, 9 H, C_3_H, C_6_H *2-Cl-Z*, C_2_H *hiN*). ^13^C-NMR δ 22.3 (γC *Lys-branches*), 23.9 (*core* γC), 25.1 (***C***H_2_-Ar *hiN*), 26.1 (*core* βC), 26.5 (*core* δC), 28.9 (δC *Lys-branches*), 31.2 (βC *Lys-branches*), 33.2, 34.7 (*core* ε, α***C***H_2_CONH), 38.1, 38.2 (εC *Lys-branches*), 39.1 (CH_2_NH *hiN*), 39.8 (*core* NH***C***H_2_***C***H_2_NH), 46.7, 49.2 [*core* α, εN-(CH_2_)_2_], 51.8 (*core* εC), 54.5 (αC *Lys-branches*), 62.3 (Ar-***C***H_2_O), 63.5 (*core* αC), 116.8 (C_4_*hiN*), 126.7, 128.8, 129.1, 129.2, 131.9, 134.1 (C_5_, C_6_, C_4_, C_3_, C_2_, C_1_*2-Cl-Z*), 134.2, 136.5 (C_2_, C_5_*hiN*), 154.9 (O-CO-NH), 170.7, 170.9 (*core* CH_2_***C***ONH), 171.2 (CONH *Lys-branches*), 171.5 (*core* CONH).

*Dendrimer**3d***. C_93_H_145_O_17_N_19_Cl_4_·6HCl, M = 2,161.8 g/mol (monoisotopic mass of unprotonated compound–1,939), white-yellow hygroscopic foam. Yield 1.0g (77%). MSLR (ESI, MeOH): 647.334 (M+3H^+^, *main signal*), 970.5 (M+2H^+^). ^1^H-NMR (400 MHz, DMSO, 298K) δ 0.9 (t, *J* 6.9 Hz, 3H, -CH_3_*dda*), 1.2–1.9 (bm, 51 H, γ, δ, βCH_2_*Lys-branches*; *core* β, γ, δCH_2_; C_2_H-C_11_H *dda*), 2.1–2.4 (bm, 10 H, C***H***_2_CONH, *core* εCH_2_), 2.5–2.9 (2 m, 8 H, α, εN-C***H***_2_-C), 2.9–3.46 (3 bm, 27 H, εCH_2_*Lys-branches*; *core* HN-C***H***_2_-C***H***_2_-NH, *core* αCH, CH_2_NH *dda*), 3.8 (m, 4 H, αCH *Lys-branches*), 5.07–5.18 (4 s, 8H, Ar-C***H***_2_O), 7.34–7.4 (m, 8 H, C_4_H, C_5_H *2-Cl-Z*), 7.42–7.55 (m, 8 H, C_3_H, C_6_H *2-Cl-Z*). ^13^C NMR δ 13.9 (C_12_*dda*), 22.4 (γC *Lys-branches*), 24.1 (*core* γC), 26.2 (*core* βC), 26.5 (C_10_, C_3_*dda*), 26.9 (*core* δC) 28.7–29.1 (C_4_-C_9_*dda*, δC *Lys-branches*), 31.1 (C_2_*dda*), 31.6 (βC L*ys-branches*), 33.3, 34.6 (*core* ε, α***C***H_2_CONH), 38.1, 38.2 (εC *Lys-branches*), 40.0 (*core* NH***C***H_2_***C***H_2_NH), 46.6, 49.1 [*core* α, εN-(CH_2_)_2_], 51.8 (*core* εC), 54.3 (αC *Lys-branches*), 62.2 (Ar-***C***H_2_O), 63.6 (*core* αC), 126.9, 128.9, 129.2, 129.3, 132.0, 134.5 (C_5_, C_6_, C_4_, C_3_, C_2_, C_1_*2-Cl-Z*), 155.2, 155.7 (O-CO-NH), 170.7, 170.9 (*core* CH_2_***C***ONH), 171.2 (*core* CONH), 171.7 (CONH *Lys-branches*).

*Dendrimer **3e***. C_86_H_124_O_17_N_20_Cl_4_·7HCl, M = 2,107.0 g/mol (monoisotopic mass of unprotonated compound–1,848), yellow-white hygroscopic foam. Yield 1.1 g (72%). MSLR (ESI, MeOH): 617 (M+3H^+^*, main signal*), 635 (M+2H^+^+Na^+^+MeOH), 925 (M+2H^+^), 952 (M+H^+^+Na^+^+MeOH) - *mass spectra obtained on MarinerTM spectrometer*, *NP* =*100, when the parameter NP* = *150 or NP* = *200 strong defragmentation occurs).* ^1^H-NMR (500 MHz, DMSO, 303K) δ 1.33, 1.43, 1.76 (3 m, 24 H, γ, δ, βCH_2_- *Lys-branches*), 1.92, 2.15 (3 m, 4 H, *core* γ, βCH_2_), 2.72, 2.82 (2 m, 8 H, *core* C***H***_2_CONH), 2.95–3.55 (4 m, 34 H, εCH_2_*Lys-branches*, α, δN-C***H***_2_-C, HN-C***H***_2_-C***H***_2_-NH and *core* δCH_2_), 3.75 (m, 4 H, αCH *Lys-branches*), 4.53 (m, 1 H, *core* αCH ), 5.07 (s, 8 H, Ar-C***H***_2_O), 7.28–7.35 (m, 8 H, C_4_H, C_5_H *2-Cl-Z*), 7.39–7.46 (m, 8 H, C_3_H, C_6_H *2-Cl-Z*), 7.90 (dd, *J* 5.7, 2.4 Hz, 1 H C_5_H *AP*), 8.60 (d, *J* 5.16 Hz, 1 H, C_4_H *AP*), 8.64 (m, C_6_H *AP*), 9.23 (s, 1 H, C_2_H *AP*). ^13^C-NMR δ 19.4 (*core* γC), 21.3 (γC *Lys-branches*), 24.5 (*core* βC), 28.5 (δC *Lys-branches*), 30.1 (βC *Lys-branches*), 29.3, 30.3 (*core* δ, α***C***H_2_CONH), 38.0 (εC *Lys-branches*), 39.9 (*core* NH***C***H_2_***C***H_2_NH), 48.1, 49.0 [*core* α, δN-(CH_2_)_2_], 51.7 (*core* δC), 52.4 (αC *Lys-branches*), 62.3 (Ar-***C***H_2_O), 63.8 (*core* αC), 126.2 (C_5_*AP*), 126.9, 128.9, 129.2, 129.3, 131.9 (C_5_, C_6_, C_4_, C_3_, C_2_*2-Cl-Z*), 134.0, 134.3 (C_6_, C_2_, *AP*), 134.4 (C_1_*2-Cl-Z*), 136.6, 138.2 (C_3_, C_4_*AP*), 155.5 (O-CO-NH), 168.3 (CONH *Lys-branches*), 168.8 (*core* CH_2_***C***ONH), 169.1 (*core* CONH).

*Dendrimer **3f***. C_91_H_130_O_17_N_20_Cl_4_·6HCl, M = 2,136.7 g/mol (monoisotopic mass of unprotonated compound–1,914), brown hygroscopic foam. Yield 1.0 g (68%). MSLR (ESI, MeOH): 583 (M-*2-Cl-Z*+3H^+^), 597.667 (M-*2-Cl-Z*+H^+^+2Na^+^), 639 (M+3H^+^), 653.667 (M+H^+^+2Na^+^*main signal*), 958 (M+2H^+^), 980 (M+2Na^+^). ^1^H-NMR (400 MHz, DMSO, 298K) δ 1.3-1.8 (bm, 28 H, γ, δ, βCH_2_*Lys-branches*; *core* β, γ, δCH_2_), 2.0-2.4 (2 m, 10 H, C***H***_2_CONH, *core* εCH_2_), 2.6-3.0 (2 m, 8 H, α, εN-C***H***_2_-C), 3.05–3.35 (bm, 26 H, εCH_2_*Lys-branches*; *core* HN-C***H***_2_-C***H***_2_-NH, CH_2_-Ar *trNH*), 3.4 (m, 3 H, -C***H***_2_-NH *trNH*, αCH), 3.85 (m, 4H, αCH *Lys-branches*), 5.1–5.2 (4 s, 8 H, Ar-C***H***_2_O), 6.96, 7.04, 7.13 (3 m, 3 H, C_1_, C_5_, C_6_*trNH*), 7.30-7.37 (bm, 9 H, C_4_H, C_5_H *2-Cl-Z*, C_7_*trNH*), 7.40-7.51 (2 bm, 8 H, C_3_H, C_6_H *2-Cl-Z*), 7.56 (1 m, 1 H, C_4_*trNH*). ^13^C-NMR δ 22.2 (γC L*ys branches*), 24.2 (*core* γC), 25.1 (*C*H_2_-Ar *trNH*), 25.7 (*core* βC), 26.6 (*core* δC), 28.8 (δC *Lys-branches*), 31.2 (βC *Lys-branches*), 33.1, 34.3 (*core* ε, α*C*H_2_CONH), 38.0, 38.1 (εC *Lys-branches*), 39.1 (-*C*H_2_-NH *trNH*), 39.8 (*core* NH*C*H_2_*C*H_2_NH), 46.7, 49.1 [*core* α, εN-(CH_2_)_2_], 51.3 (*core* εC), 54.3 (αC *Lys-branches*), 62.2 (Ar-*C*H_2_O), 63.6 (*core* αC), 111.0, 111.7, 117.8, 117.9, 120.6, 122.1 (C_7_, C_2_, C_4_, C_5_, C_6_, C_1_*trNH*), 126.7 (C_5_*2-Cl-Z*), 127.0 (C_3_*trNH*), 128.8, 129.0, 129.1, 131.8, 134.1 (C_6_, C_4_, C_3_, C_2_, C_1_*2-Cl-Z*), 136.1 (C_8_*trNH*), 155.2 (O-CO-NH), 170.7, 170.9 (*core* CH_2_*C*ONH), 171.5 (CONH *Lys-branches*), 171.8 (*core* CONH).

*Dendrimer **3g***. C_86_H_127_O_17_N_21_Cl_4_·7HCl, M = 2,124.1 g/mol (monoisotopic mass of unprotonated compound–1,865), yellow-white hygroscopic foam. Yield 1.0 g (76%). MSLR (ESI, MeOH): 566.667 (M-*2-Cl-Z*+3H^+^), 622.667 (M+3H^+^, *main signal*), 933.5 (M+2H^+^). ^1^H-NMR (400 MHz, DMSO, 298K) δ 1.3–1.7 (bm, 28 H, γ, δ, βCH_2_*Lys-branches*; *core* β, γ, δCH_2_), 2.14–2.4 (2 m, 10 H, C***H***_2_CONH, *core* εCH_2_), 2.6-2.9 (3 m, 10 H, α, εN-C***H***_2_-C, CH_2_-Ar *hiN*), 3.1-3.4 (2 bm, 27 H, εCH_2_*Lys-branches*, *core* HN-C***H***_2_-C***H***_2_-NH, *core* αCH, CH_2_NH *hiN*), 3.93 (m, 4 H, αCH *Lys branches*), 5.08–5.19 (4 s, 8 H, Ar-C***H***_2_O), 6.77 (s, 1 H, C_4_H *hiN*), 7.31–7.37 (m, 8 H, C_4_H, C_5_H *2-Cl-Z*), 7.4–7.5 (m, 9 H, C_3_H, C_6_H *2-Cl-Z*, C_2_H *hiN*). ^13^C-NMR δ 22.4 (γC *Lys-branches*), 23.8 (*core* γC), 25.0 (***C***H_2_-Ar *hiN*), 26.2 (*core* βC), 26.8 (*core* δC), 28.9 (δC *Lys-branches*), 31.1 (βC *Lys-branches*), 33.2, 34.5 (*core* ε, α***C***H_2_CONH), 38.1, 38.2 (εC *Lys-branches*), 39.1 (CH_2_NH *hiN*), 39.9 (*core* NH***C***H_2_***C***H_2_NH), 46.7, 49.2 [*core* α, εN-(CH_2_)_2_], 51.6 (*core* εC), 54.4 (*core* αC), 62.2 (Ar-***C***H_2_O), 63.3 (*core* αC), 116.8 (C_4_*hiN*), 126.7, 128.8, 129.1, 129.2, 131.8, 134.1 (C_5_, C_6_, C_4_, C_3_, C_2_, C_1_*2-Cl-Z*), 134.2, 136.5 (C_2_, C_5_*hiN*), 155.1 (O-CO-NH), 170.7, 170.8 (*core* CH_2_***C***ONH), 171.1 (CONH *Lys-branches*), 171.4 (*core* CONH).

*Dendrimer **3h***. C_93_H_145_O_17_N_19_Cl_4_·6HCl, M = 2,161.8 g/mol (monoisotopic mass of unprotonated compound–1,939), white-yellow hygroscopic foam. Yield 1.1 g (70%). MSLR (ESI, MeOH): 647.334 (M+3H^+^, *main signal*), 970.5 (M+2H^+^). ^1^H-NMR (400 MHz, DMSO, 298K) δ 0.9 (t, *J* 6.9 Hz, 3 H, -CH_3_*dda*), 1.1–1.9 (bm, 51 H, γ, δ, βCH_2_*Lys-branches*, *core* β, γ, δCH_2_, C_2_H-C_11_H *dda*), 2.1–2.4 (bm, 10 H, C***H***_2_CONH, *core* εCH_2_), 2.6–2.9 (2 m, 8 H, α, εN-C***H***_2_-C), 2.9–3.46 (3 bm, 27 H, εCH_2_*Lys-branches*, *core* HN-C***H***_2_-C***H***_2_-NH, *core* αCH, CH_2_NH *dda*), 3.9 (m, 4 H, αCH *Lys-branches*), 5.05–5.16 (4 s, 8 H, Ar-C***H***_2_O), 7.33–7.4 (m, 8 H, C_4_H, C_5_H *2-Cl-Z*), 7.45–7.55 (m, 8 H, C_3_H, C_6_H *2-Cl-Z*). ^13^C-NMR δ 14.0 (C_12_*dda*), 22.4 (γC *Lys*), 24.5 (γC *core*), 26.4 (βC *core*), 26.5 (C_10_, C_3_*dda*), 28.7–29.1 (C_4_-C_9_*dda*, δC *Lys*), 31.1 (C_2_*dda*), 31.5 (βC *Lys*), 33.3, 34.6 (δ, α***C***H_2_CONH *core*), 38.1, 38.2 (εC *Lys-branches*), 39.9 (NH***C***H_2_***C***H_2_NH *core*), 46.7, 49.1 [α, δN-(CH_2_)_2_*core*], 51.6 (δC *core*), 54.2 (αC *Lys*), 62.4 (Ar-***C***H_2_O), 63.6 (αC *core*), 126.9, 128.9, 129.2, 129.3, 132.0, 134.5 (C_5_, C_6_, C_4_, C_3_, C_2_, C_1_*2-Cl-Z*), 155.4, (O-CO-NH), 170.6, 170.8 (CH_2_***C***ONH *core*), 171.3 (CONH *core*), 171.7 (CONH *Lys-branches*).

### 3.3. Antibacterial Susceptibility Testing

The bacteria *Staphylococcus aureus* ATCC 25923, *Staphylococcus aureus* ATCC 43300,*Escherichia c*oli ATCC 25922 and *Pseudomonas aeruginosa* ATCC 27853 were cultivated on tryptone-soy agar (TSA; Oxoid) for 24 h at 37 °C. Broth microdilution susceptibility tests were performed as described in the Committee Laboratory Standards (CLSI) reference method M07-A8 [41]. Bacteria inocula (5 µL) containing 10^6^ CFU/mL were used. The final concentration of dendrimers ranged from 256 to 2 µg/mL, polymyxin B and penicillin G from 8 to 0,15 µg/mL (or 5.8 to 0.1 µM and 21.5 to 0.4 µM, respectively for polymyxin B and penicillin G) were the reference compounds. The plates were incubated at 35 °C and were read after 18 or 24 h depending on the bacterial strain. Minimal Inhibitory Concentrations (MICs) were defined as the lowest drug concentration that reduced the growth by 100%.

The effect of salt concentration on the antimicrobial activity of two dendrimers was tested by determining the MICs under a variety of cations concentrations. CAMHB used in the assay was altered by the addition of salt. NaCl or KCl were added to the media to a final concentrations of 10, 50, 100, 200, 500 mM, wheareas CaCl_2_ or MgCl_2_ were added to the media to a final concentrations of 0.5, 1.5, 2.5, 5.5, 10.5, and 20.5 mM. Additionally, similar test was performed using MHB (without Ca^2+^, Mg^2+^) for divalent cations Ca^2+^ and Mg^2+^. Experiment was performed in triplicate.

### 3.4. Hemolysis Assay

Dendrimer-induced hemolysis was measured as previously reported [42]. Briefly, human red blood cells obtained from healthy volunteers, were suspended in phosphate buffered saline (PBS, pH 7.4). Prepared suspension of 1% hematocrit was incubated with serial concentration of dendrimers for 30 min at 23 °C. After centrifugation (4,000 rpm, 5 min) the absorbance of supernatant was measured at 540 nm (Spectrostar Omega, BMG Labtech, Ortenberg, Germany). A value of 100% hemolysis was determined by incubation of erythrocytes with double-distilled water (30 min at 23 °C). In a control experiment, cells were incubated in buffer without peptide and absorbance at 540 nm value was used as a blank:


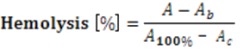


A: Absorbance of the samples incubated with dendrimers;

A_b_: Absorbance of the blank samples;

A_100%_: Absorbance of the reference;

A_c_: Absorbance of red blood cells in PBS, hematocrit 1%.

### 3.5. Circular Dichroism Spectroscopy

The CD spectra were recorded on a JASCO J-815 spectropolarimeter and were smoothened by the Savitzky-Golay method. The peptide concentrations for the spectra reported here were in the 79 to 89 µM range, in a cell of 0.1 cm path length or *ca.* 80 µM in salt titration experiments.

### 3.6. Mass Spectrometry Measurements

Electrospray mass spectra were recorded on an API 365 triple quadrupole mass spectrometer (Applied Biosystems, Foster City, CA, USA). The mass spectrometer was equipped with a TurboIonSpray^TM^ electrospray ion source operated in the standard positive ESI mode, *i.e.*, without additional drying gas. Self-complementary denrimer and phospholipid stock solutions were prepared in methanol at concentration of 1.4 mM. When solubility in methanol was too low, chloroform was added in as small amounts as possible. Sample mixtures, consisting of dendrimer at 15 µM and phospholipid at 30 µM in methanol, were used for mass analysis. The analyte solutions were infused at 10 µL/min directly into the mass spectrometer. The ion source parameters were optimized to obtain the highest possible abundance of the complexes and were adjusted as follows: ion spray voltage (IS) 4.5 kV, declustering potential (DP) 20, focusing potential (FP) 200, entrance potential (EP) 10.

The collisional dissociation spectra were recorded on a 4000 QTrap, Linear Ion trap triple quadrupole mass spectrometer (AB Sciex), equipped with an electrospray ion source. The fragmentation spectra were collected in the enhanced product ion (EPI) mode with a fixed LIT fill time of 20 ms. The ion source parameters were as follows: spray voltage (IS) 5 kV, declustering potential (DP) 60. In the CID experiments, nitrogen was used as the collision gas (pressure ca. 4.5 · 10^−5^ Torr) and the collision energy was varied in the range of 5 eV to 40 eV (laboratory frame). The collisional experiments were repeated on SYNAPT G2 HDMS (Waters) Quadrupole-Ion-Mobility-Time-of-Flight mass spectrometer. The CID spectra were recorded as a function of transfer collision energy, which was varied in the range of 0–30 eV (laboratory frame). In this set of experiments argon was used as a collision gas. The ion source parameters were as follows: the spray voltage: 2.5 kV, the source temperature: 80 °C, the sampling cone voltage: 11 V, the desolvation gas flow rate: 224 L/h, the desolvation temperature: 150 °C. The break-down graphs obtained in this part of experiments are shown in supporting information – Figure S8. For presented plots, each point represents the average of three measurements (each measurement averaged over 20 scans). There was little variation (2.5%) in the relative product ion abundances from measurements.

## 4. Conclusions

In summary, we have demonstrated that the adopted synthetic strategy provides the opportunity to synthesize branched peptides with independent variation of three residues by robust and simple chemistry. This involves perfect control of chemical character and relative orientations of lipophilic and cationic residues, arm length, as well as localization and number of positive charges. This option is particularly useful for the preparation of bioactive peptide dendrimers with amphiphilic surfaces and multiple functionality. Brief antimicrobial testing demonstrated that the dendrimers designed in the present work expressed either high potency against Gram-positive *S. aureus* or a broader activity against *S. aureus* and Gram-negative *E. coli* and *P. aeruginosa* strains. In particular, dendrimer **3d** showed interesting properties of low hemotoxicity and strong activity against MRSA *S. aureus* and Gram-negative *P. aeruginosa.* Thus, these dendrimeric peptides exhibited antimicrobial profiles and potency in the range of that of linear natural antimicrobial peptides. Antimicrobial susceptibility against *E. coli* ATCC 25922 tested for two diastereoisomeric compounds showed their high activity in presence of physiological concentrations of monovalent (Na^+^ and K^+^) cations and a lower activity in the presence of divalent (Mg^2+^ and Ca^2+^) cations. Circular dichroism (CD) studies confirmed that dendrimer conformations are solvent, ionic strength and concentration dependent. 

Gas phase studies on interactions of the selected dendrimers and model DMPG and DMPC phospholipids demonstrated that mass spectrometry can be used to verify details of the dendrimer/phospholipid recognition process driven by electrostatic interactions taking place at the biological interfaces. In particular, observed correlation between hemotoxicity and differences in relative dissociation energy of the complexes formed between dendrimers and anionic (DMPG) as well as neutral (DMPC) phospholipids (∆CID_50_) offer information on affinity and selectivity of the studied dendrimers towards model phospholipids in relation to the chemical structure of dendrimers and phospholipids.

Both circular dichroism and mass spectrometry studies evidenced that dendrimers of *N*^α^- and *N*^ε^-series have different conformations in solution and different affinity to model phospholipids, what might influence detailed microbicidal mechanism.

Recently, studies of dendrimers have become one of the most fruitful areas in biomedical sciences. Their most exploited property is polyvalency, *i.e.*, the ability to deliver multiple ligands to the receptor site. However, the full potential of these versatile molecules as mimetics of biologically active systems remains to be fully explored. As compared to antibiotics used in the clinic, the “unknown” to microbial world branched structure of dendrimers is a big advantage and makes resistance development significantly more difficult.

## Supplementary Materials

Supplementary materials can be accessed at: http://www.mdpi.com/1420-3049/18/6/7120/s1.
