# Supplementary material for: Novel Antimicrobial Peptide Dendrimers with Amphiphilic Surface and Their Interactions with Phospholipids — Insights from Mass Spectrometry
Source: Molecules. 2013 Jun 18;18(6):7120–44. doi: 10.3390/molecules18067120 (PMC6270063; doi:10.3390/molecules18067120)

Supplementary Materials

Figure S1. Molar ellipticity of dendrimers **3c** measured in MeOH and H_2O at different concentrations.

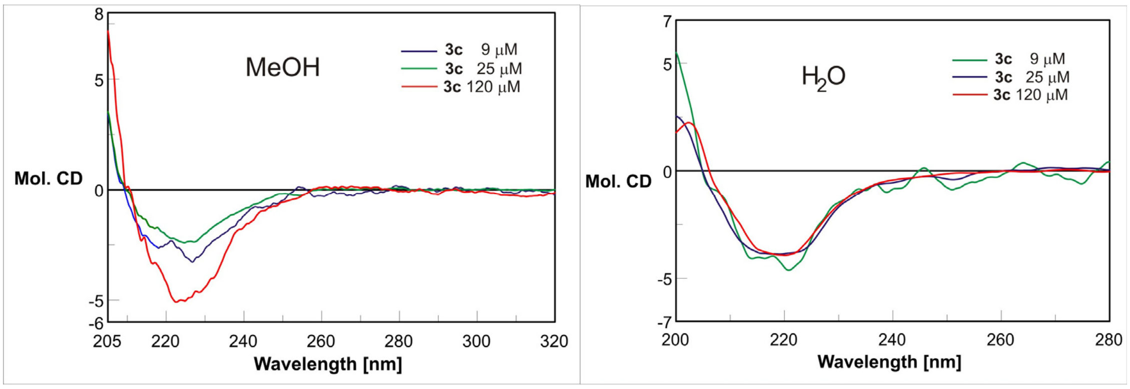

Figure S2. Molar ellipticity of dendrimers **3a-3h**, measured in MeOH and selected dendrimers **3b**, **3f** in presence of 5, 10, 20-fold molar excess of $CaCl_2$, peptide concentration ca 80 µM.

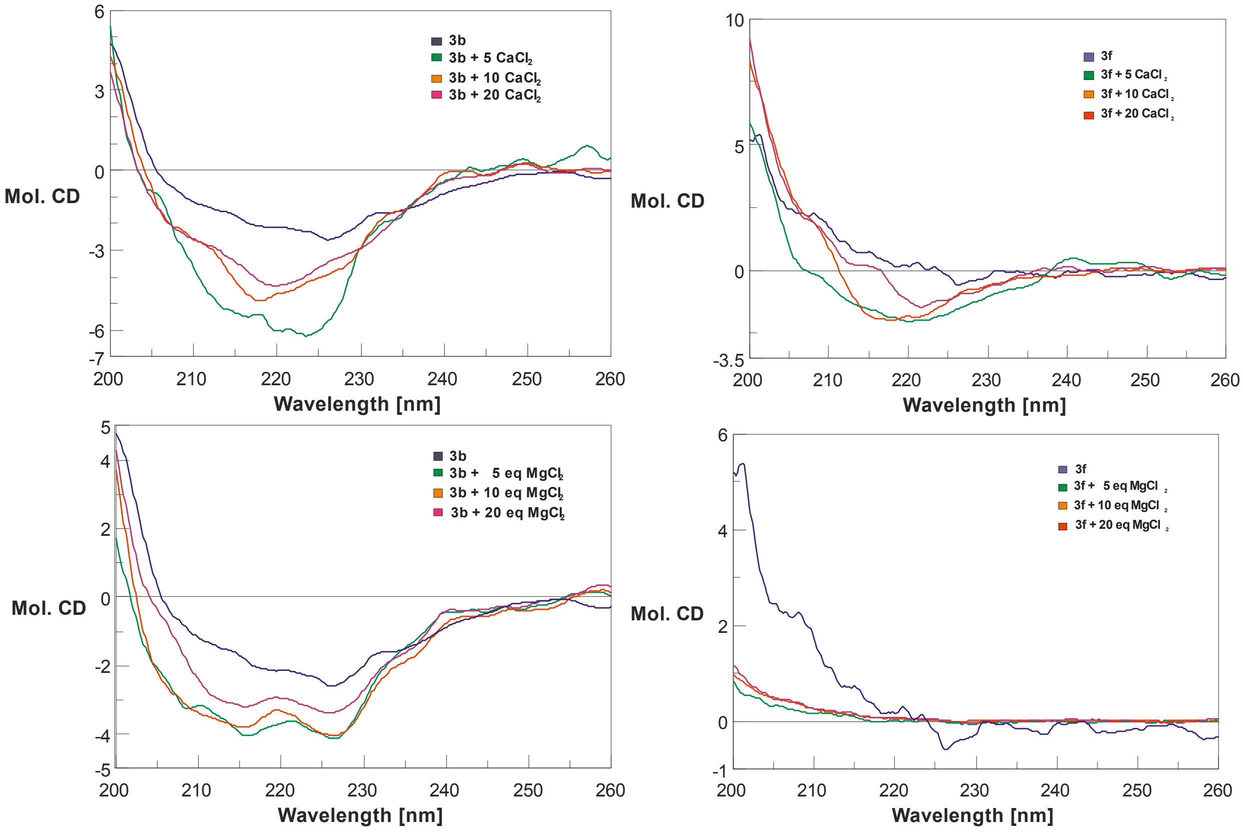

Figure S3. ESI MS spectra for 1:2 mixture of dendrimer **3c** with DMPC.

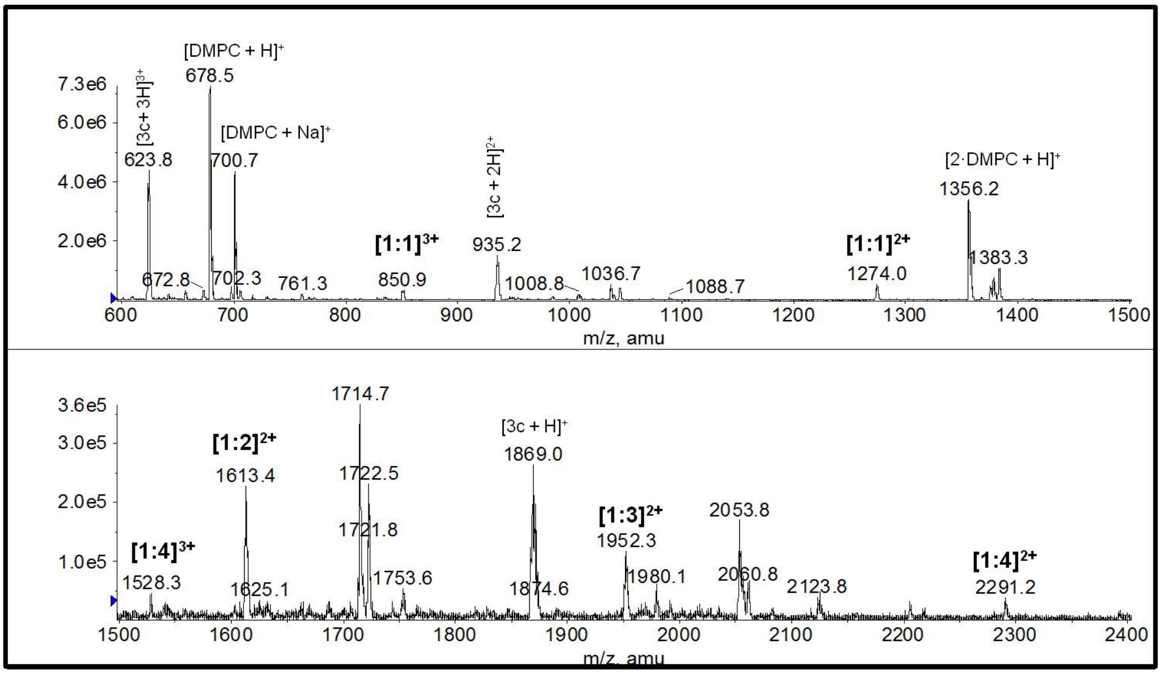

Figure S4. ESI MS spectra for 1:2 mixture of dendrimer **3h** with DMPC.

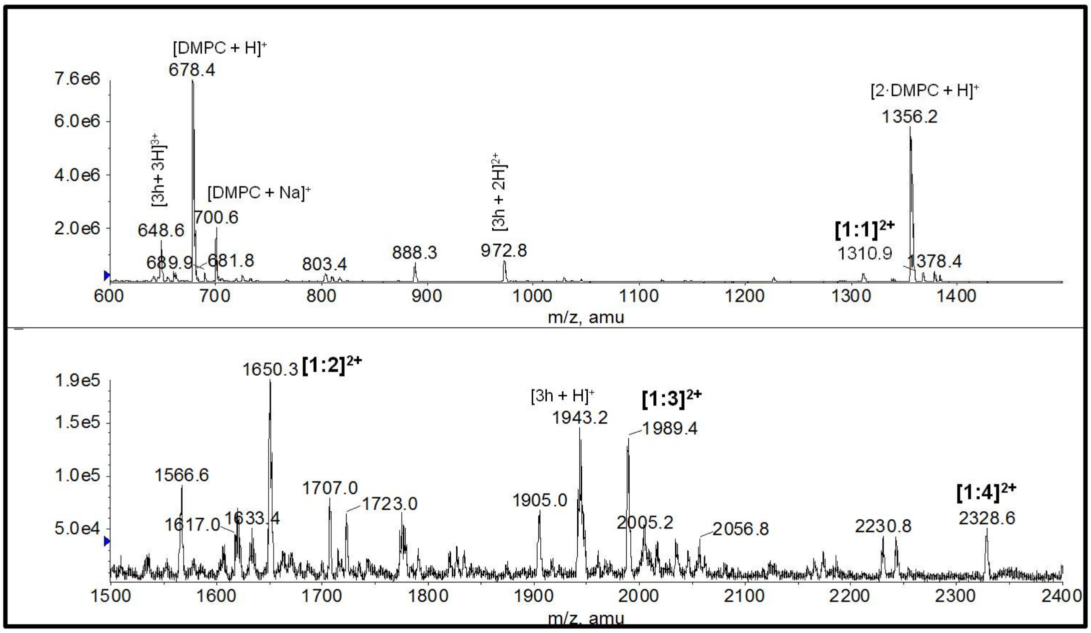

Figure S5. ESI MS spectra for 1:2 mixture of dendrimer **3c** with DMPG.

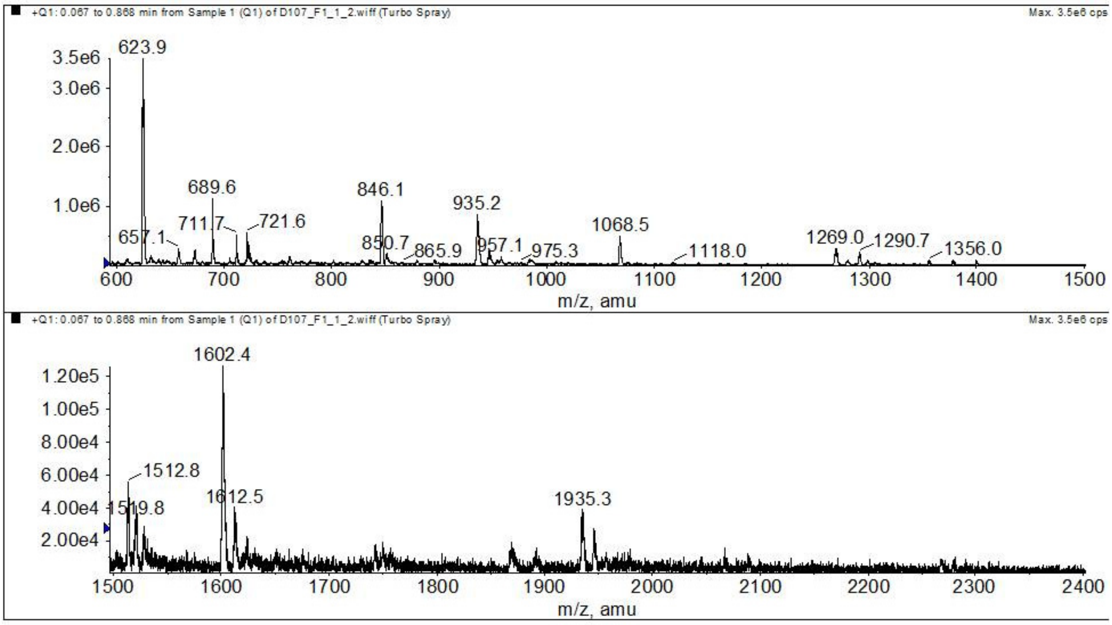

Figure S6. ESI MS spectra for 1:2 mixture of dendrimer **3h** with DMPG.

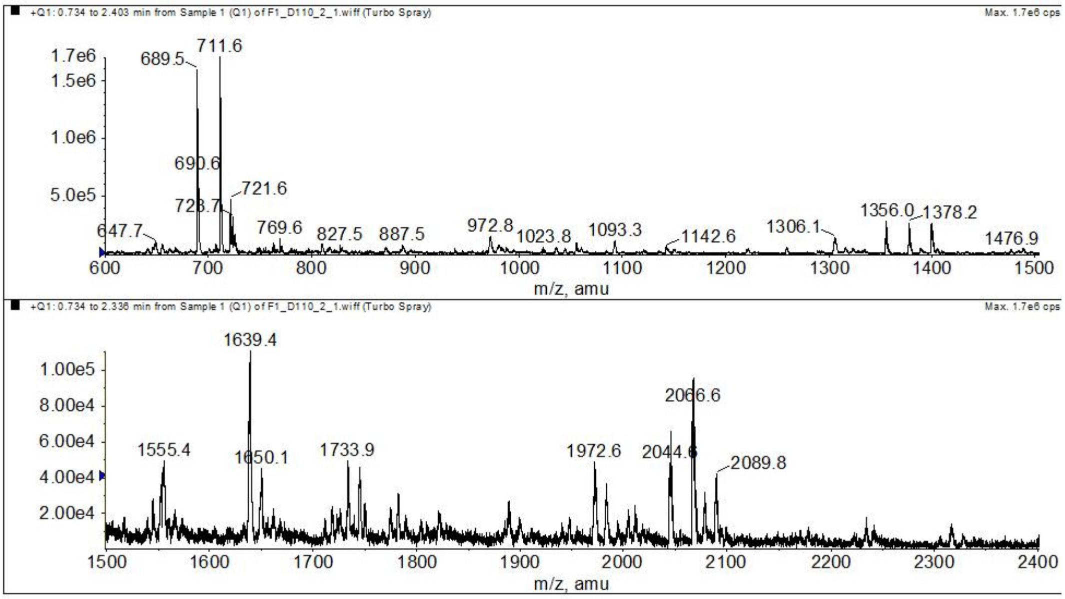

Figure S7. Collision-induced dissociation (CID) spectra of doubly charged complexes of (**a**) DMPG/**3a** and (**b**) DMPC/**3a** complexes. The collision gas was nitrogen; collision energy was 15 eV (in the laboratory frame).

a)

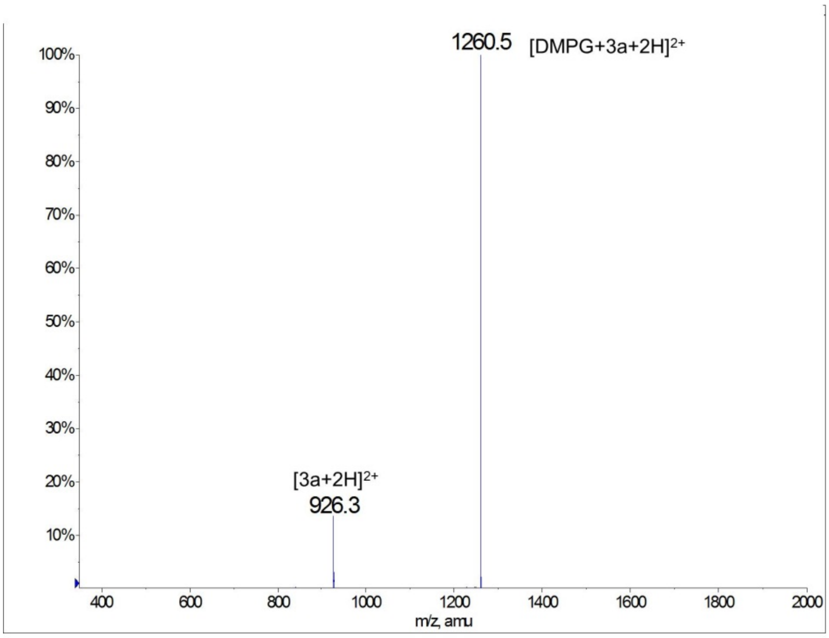

b)

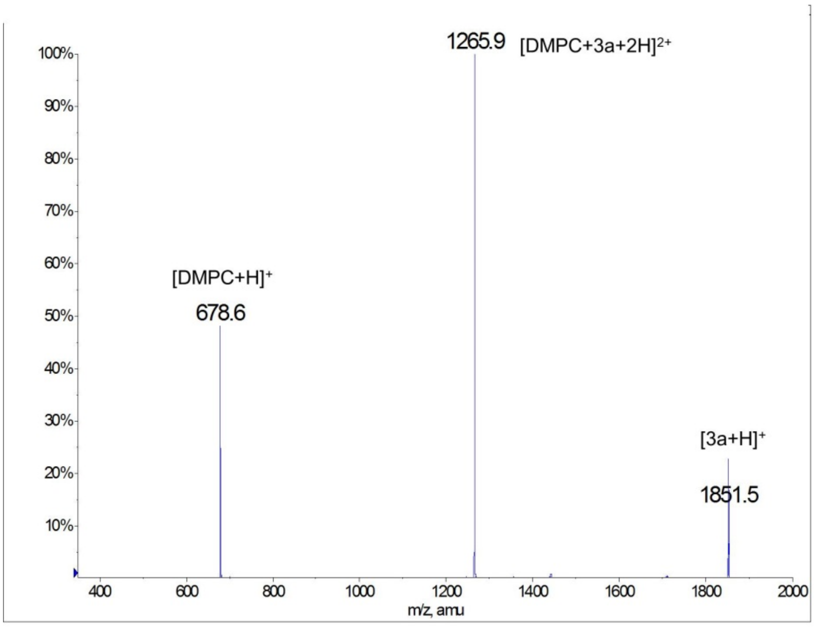

Figure S8. Dissociation efficiency curves (experiments repeated on SYNAPT G2 HDMS) of dendrimer/phospholipid $[1:1]^{2+}$ complexes: (**a**) DMPG/**3h** (red square), DMPG/**3c** (green triangle), DMPG/**3a** (blue square), DMPG/**3d** (cross) and (**b**) DMPC/**3h** (red square), DMPC/**3c** (green triangle), DMPC/**3a** (blue square), DMPC/**3d** (cross). The *y* and *x* axis represents the relative intensity of a complex in proportion to the sum of the intensities of the fragment ions and center-of mass collision energy under single collision conditions, respectively.

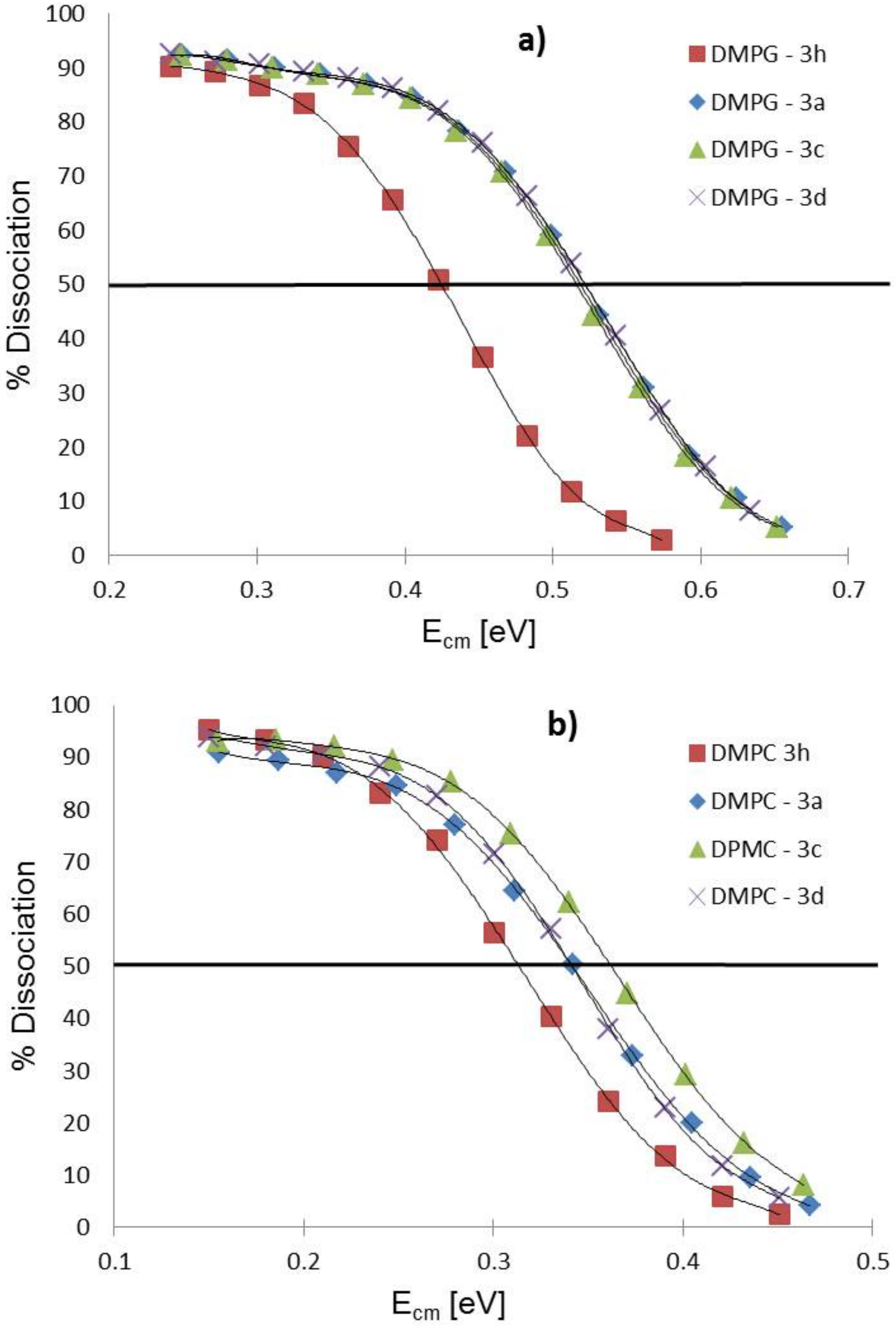

Supplement: Supplementary file 1 [file molecules-18-07120-s001.pdf]
